# Hypoxia controls expression of kidney-pathogenic *MUC1* variants

**DOI:** 10.26508/lsa.202302078

**Published:** 2023-06-14

**Authors:** Stephanie Naas, René Krüger, Karl Xaver Knaup, Julia Naas, Steffen Grampp, Mario Schiffer, Michael Wiesener, Johannes Schödel

**Affiliations:** 1 Department of Nephrology and Hypertension, Uniklinikum Erlangen und Friedrich-Alexander-Universität Erlangen-Nürnberg, Erlangen, Germany; 2 Center for Integrative Bioinformatics Vienna (CIBIV), Max Perutz Labs, University of Vienna and Medical University of Vienna, Wien, Austria

## Abstract

The interplay between genetic and environmental factors influences the course of chronic kidney disease (CKD). In this context, genetic alterations in the kidney disease gene *MUC1* (Mucin1) predispose to the development of CKD. These variations comprise the polymorphism rs4072037, which alters splicing of MUC1 mRNA, the length of a region with variable number of tandem repeats (VNTR), and rare autosomal-dominant inherited dominant-negative mutations in or 5′ to the VNTR that causes autosomal dominant tubulointerstitial kidney disease (ADTKD-*MUC1*). As hypoxia plays a pivotal role in states of acute and chronic kidney injury, we explored the effects of hypoxia-inducible transcription factors (HIF) on the expression of *MUC1* and its pathogenic variants in isolated primary human renal tubular cells. We defined a HIF-binding DNA regulatory element in the promoter-proximal region of *MUC1* from which hypoxia or treatment with HIF stabilizers, which were recently approved for an anti-anemic therapy in CKD patients, increased levels of wild-type *MUC1* and the disease-associated variants. Thus, application of these compounds might exert unfavorable effects in patients carrying *MUC1* risk variants.

## Abstract (precis)

Hypoxia or pharmacological treatment with novel HIF stabilizers promotes the expression of *MUC1* genetic variants that predispose to the development of chronic kidney disease in renal tubular cells.

## Introduction

Genetic and environmental factors shape the development and progression of chronic kidney disease (CKD) leading to extremely heterogeneous clinical courses (Susztak, 2014; Obrador et al, 2017). Exploring the underlying mechanisms for this heterogeneity is a major task in kidney research to identify people at risk and to optimize therapy for individual CKD patients. Genome-wide association studies (GWAS) have uncovered that genetic variation at the chromosome 1q21 locus including the *MUC1* gene associates with various kidney-related traits including urate levels, urea levels, estimated glomerular filtration rate (eGFR), and albuminuria (Okada et al, 2012; Kottgen et al, 2013; Xu et al, 2018; Morris et al, 2019; Teumer et al, 2019; Tin et al, 2019). In this respect, the common polymorphism rs4072037 (T > C; minor allele frequency in 1,000 Genomes Project phase 3: 0.371) within the second exon of *MUC1* has been worked up and the T allele has been shown to introduce an alternative splice site leading to expression of MUC1 transcripts with a 27-nucleotide in-frame deletion in various tissues including the kidney (Ligtenberg et al, 1991; Imbert et al, 2006; Xu et al, 2018). Expression levels of these alternative transcripts are more related to eGFR than are the total expression levels of MUC1 implying that they are causal in generating the GWAS-detected effects (Xu et al, 2018). In addition, the length of a region with variable number of tandem repeats (VNTR), which contains 20–125 repeats of a 60-bp sequence in exon 2 of the *MUC1* gene, has recently been associated with kidney traits (Mukamel et al, 2021). Longer VNTR alleles (>55 repeats) associate with increased serum urea, serum urate, and chronic interstitial nephritis and weakly with reduced eGFR and lower hemoglobin levels in the UK Biobank cohort (Mukamel et al, 2021). Whereas these genetic variants may affect millions of individuals with a putative disease-modulating effect, rare dominant-negative mutations within or 5′ to the VNTR region leading to expression of a short frameshift protein of MUC1 (MUC1-fs) cause autosomal dominant tubulointerstitial kidney disease (ADTKD-*MUC1*). This MUC1-fs mutation provokes a proteinopathy in which the MUC1-fs protein accumulates in the early secretory pathway of epithelial cells where it is thought to exert its harmful effect via dysregulation of the unfolded protein response pathway (Dvela-Levitt et al, 2019). Ultimately, expression of MUC1-fs protein leads to tubular cell death, fibrosis and reduced kidney function often requiring renal replacement therapy (Olinger et al, 2020).

In the healthy human kidney, *MUC1* is expressed in distal tubule segments including the thick ascending limb (TAL) suggesting that effects on renal disease traits might be generated from this part of the nephron (Heyderman et al, 1979; Knaup et al, 2018; Zivna et al, 2018). Interestingly, in mouse models of ischemic acute kidney injury, *MUC1* expression was also detected in proximal tubule cells (Al-Bataineh et al, 2021). Under physiological conditions, the MUC1 extracellular domain functions together with other proteins of the mucin family as a protective barrier in epithelia against microbial and environmental damage (Wagner et al, 2018; Devuyst et al, 2019). The expression of *MUC1* is often dysregulated in epithelial cancers suggesting that many factors may influence *MUC1* regulation. In this respect, *MUC1* expression has been associated with hypoxia signaling (Aubert et al, 2009). Hypoxia-inducible transcription factors (HIF) are the main effectors of oxygen-sensing and transcriptionally regulate many hundred genes to adapt the cell and the body to low oxygen concentrations (Semenza, 2012; Schodel & Ratcliffe, 2019). In the kidney, a hypoxic microenvironment leading to stabilization of HIF is fundamentally involved in the biology of acute and chronic diseases (Haase, 2017; Schodel & Ratcliffe, 2019). Moreover, dysfunction of this pathway causes renal anemia through insufficient induction of erythropoietin (EPO) by HIF-2α in interstitial cells of the kidney (Gruber et al, 2007; Schodel & Ratcliffe, 2019). Therefore, pharmacological compounds have recently been introduced to clinical practice in many countries to stimulate EPO production by stabilizing HIF in the diseased kidney of CKD patients before and on dialysis (Maxwell & Eckardt, 2016; Chen et al, 2019; Chertow et al, 2021; Singh et al, 2021). These substances selectively interfere with the enzymatic activity of prolyl hydroxylase domain proteins thereby inhibiting hydroxylation of proline residues and consequently preventing proteasomal degradation of the HIF-α subunit (Yeh et al, 2017). Though the aim of using these compounds is to induce HIF-2α and EPO production in a specific subpopulation of interstitial cells of the kidney, these compounds are likewise capable of generally stabilizing HIF-1α, for example, in tubular cells of the kidney (Rosenberger et al, 2002; Schley et al, 2012).

In this work, we establish a link between regulation of *MUC1* expression including the deleterious variants and HIF signaling in renal tubular cells.

## Results

**Figure 1.**
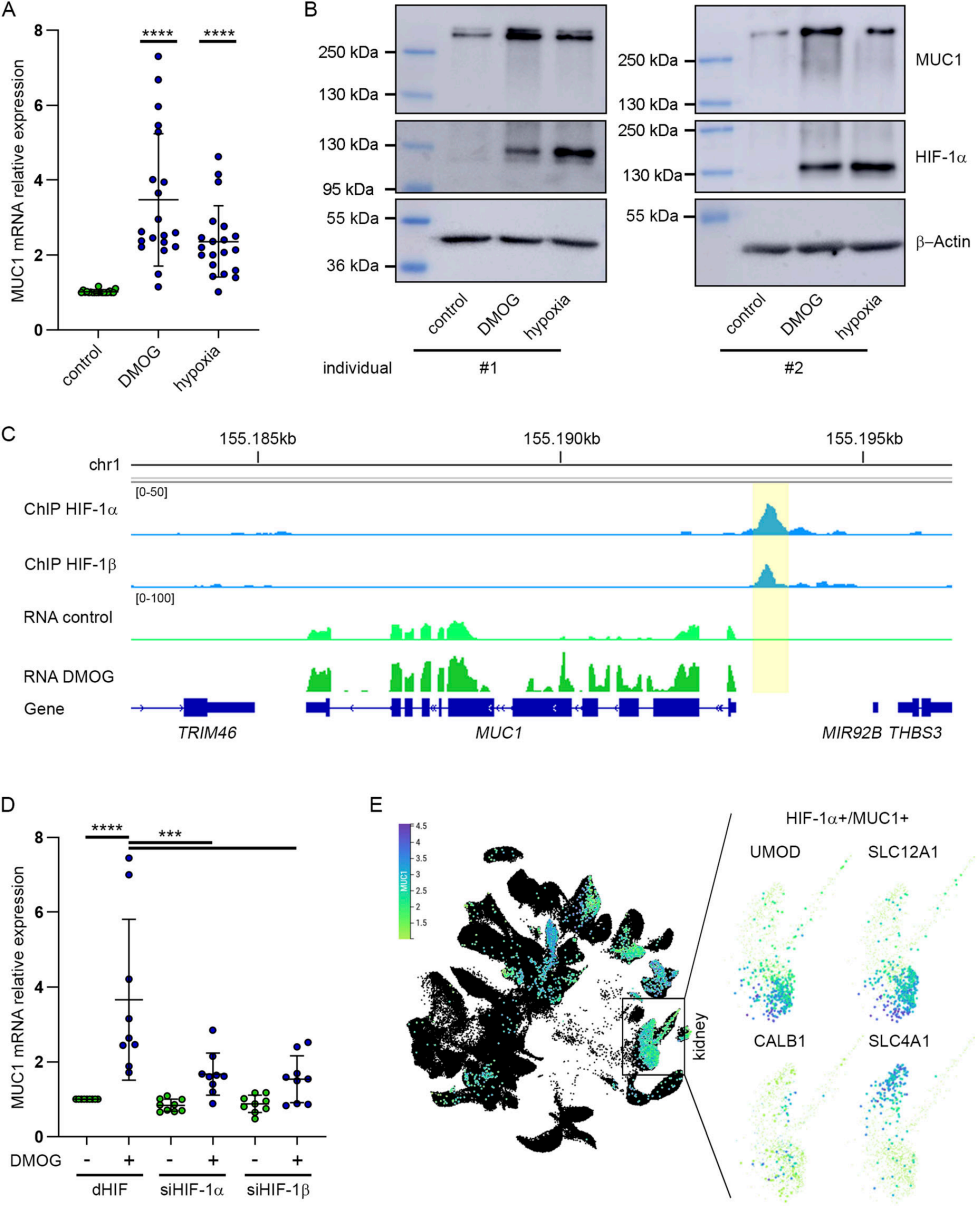
HIF regulates *MUC1* expression in primary tubular cells (PTC). **(A)** Expression qRT-PCR analysis of MUC1 mRNA in PTC treated with 1 mM DMOG or 1% O_2_ for 16 h compared to control condition (20.9% O_2_; n = 20 independent individuals). Values were normalized to expression levels of the housekeeping gene *HPRT*. One sample *t* test of MUC1 mRNA expression levels in treatment conditions versus a theoretical value of one representing no regulation, ****P < 0.0001. Bars indicate mean fold change ± SD. **(B)** Immunoblot for MUC1, HIF-1α, and β-actin from lysates of PTC from two different individuals incubated with 1 mM DMOG or cultured in 1% O_2_ (hypoxia) for 16 h as indicated. **(C)** ChIP-seq and RNA-seq tracks from PTC at the *MUC1* locus. ChIP-seq tracks (blue: DMOG-treated cells) reveal HIF-1α and HIF-1β-binding signals (highlighted in yellow box) within the promoter-proximal region of *MUC1*. RNA-seq tracks (light green: control; green: DMOG-treated cells) reveal increased expression of MUC1 mRNA in PTC treated with 1 mM DMOG for 16 h when compared with control. **(D)** Expression qRT-PCR analysis for MUC1 mRNA from PTC depleted for HIF-1α or HIF-1β using siRNA. Values were compared with results from cells treated with non-targeting control siRNA (drosophila HIF, dHIF). Cells were exposed to 1 mM DMOG or control condition for 16 h (n = 9 independent experiments from PTC isolated from nine different individuals). Two-way ANOVA followed by pairwise comparison, ***P < 0.001, ****P < 0.0001. Bars indicate mean ± SD. **(E)** Uniform Manifold Approximation and Projection (UMAP) plot of single-cell mRNA-seq data from all major adult human organs comprising 335,199 cells in total (data derived from Han et al [2020]). Scale bar shows normalized UMI counts in (ln [CPM/100 + 1]). Cells expressing MUC1 and HIF-1α mRNA were highlighted and coloured based on expression of MUC1 (n = 27,011 double-positive cells). Dark blue indicates high levels of MUC1 mRNA. Zoom depicts a major cell cluster that was assigned to kidney tissue. Analysis for co-expression within these clusters using marker gene transcripts for the thick ascending limb (UMOD and SLC12A1) and marker transcripts for more distal tubular segments (CALB1 and SLC4A1) revealed overlap of MUC1 and HIF-1α double-positive cells in these segments. Source data are available for this figure.

**Figure S1.**
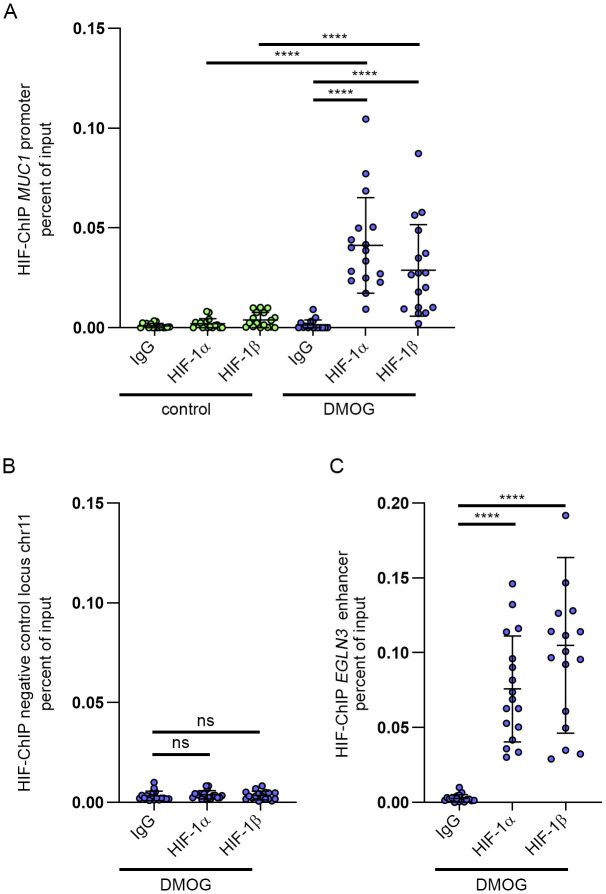
HIF is recruited to the *MUC1* promoter-proximal regulatory element in primary tubular cells exposed to HIF-stabilizing agent DMOG. **(A)** HIF ChIP-qRT-PCR for the *MUC1* promoter-proximal regulatory DNA element in samples derived from 17 independent primary tubular cell isolates exposed to 1 mM DMOG or untreated control conditions for 16 h. Values were normalized to qRT-PCR results obtained from respective input DNA. Bars indicate mean values ± SD. Two-way ANOVA followed by pairwise comparison, ****P < 0.0001. **(B)** HIF ChIP-qRT-PCR for a negative control locus at chromosome 11 performed in the DMOG-treated subset of samples used in (A). Values were normalized to qRT-PCR results obtained from respective input DNA. One-way ANOVA followed by pairwise comparison. Ns, not significant. **(C)** HIF ChIP-qRT-PCR for the EGLN3 enhancer known to constitute a robust HIF-binding site conducted in same samples as (B). Values were normalized to qRT-PCR results obtained from input DNA. Bars indicate mean values ± SD. One-way ANOVA followed by pairwise comparison. ****P < 0.0001.

**Figure S2.**
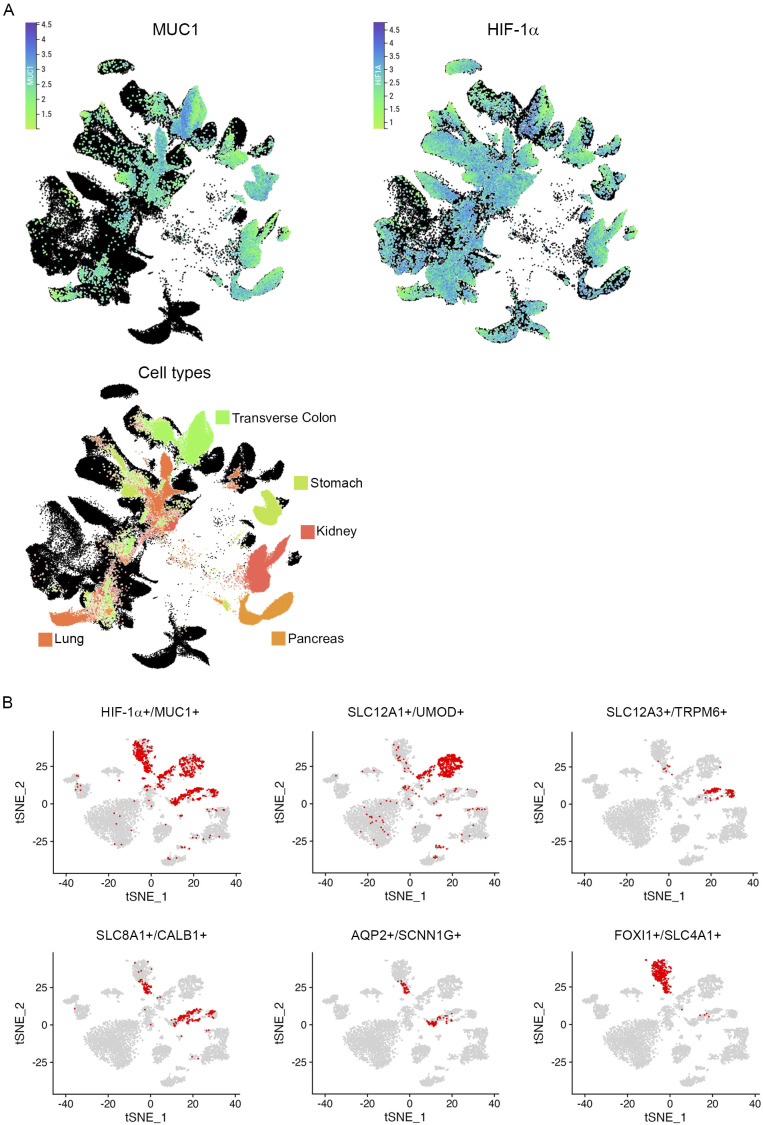
Thick ascending limb of loop of Henle and more distal tubular segments co-express MUC1 and HIF-1α mRNA. **(A)** Uniform Manifold Approximation and Projection (UMAP) plot of single-cell mRNA-seq data from all major adult human organs (data are from Han et al [2020]). Same data as in main Fig 1E and Table S1. Cells expressing MUC1 (first UMAP, left) or HIF-1α (second UMAP, right) were highlighted and coloured based on the respective expression level. Scale bars show normalized UMI counts in (ln [CPM/100 + 1]). Dark blue indicates high mRNA expression level. Third UMAP: Cell types expressing high levels of *MUC1* were marked. Cells are colour-coded according to assigned tissue. **(B)** *t*-SNE (*t*-distributed stochastic neighbor embedding) plot of single-cell RNA-seq data acquired in adult kidneys (data are from Zhang et al [2021]). Same data as in Table S2. Comparison of double-positive cells (HIF-1α+/MUC1+) with cells expressing the indicated marker genes reveal distal tubular origin. Cells were denoted “positive” if ≥ raw 1 UMI read(s) of the respective gene was counted. Cells co-expressing SLC12A1 and UMOD transcripts originate from the thick ascending limb. Distal convoluted cells show expression of SLC12A3 and TRPM6 mRNA, whereas cells declared as connecting tubules express SLC8A1 and CALB1 transcripts. Principal cells typically show expression of AQP2 and SCNN1G. Double positivity for FOXI1 and SLC4A1 mRNA mark intercalated cells. Respective cells are highlighted in red.

**Figure S3.**
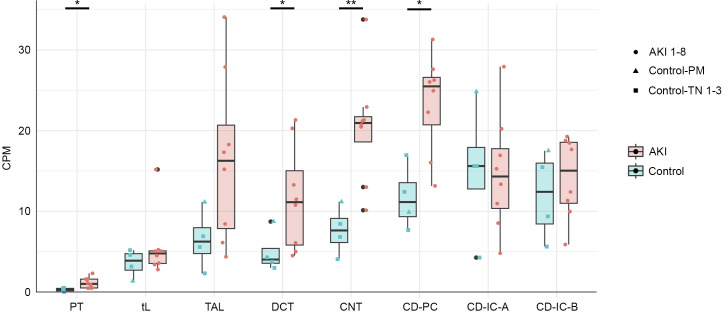
Single-nucleus RNA-seq data reveal increased *MUC1* expression in distal tubular cells from acutely injured kidneys. In cells from non-injured control kidneys (green boxplots), MUC1 mRNA is expressed predominantly in distal tubular cells including the thick ascending limb (TAL), the distal convoluted tubule (DCT), the connecting tubule (CNT), and cells of the collecting duct (CD-PC/IC-A/IC-B, collecting duct principal/intercalated cell types A and B). In acute kidney injury (AKI, red boxplots), up-regulation of *MUC1* expression can be detected especially in these distal tubular segments (TAL, DCT, CNT) (Hinze et al, 2022). PT, proximal tubule; tL, thin limb. Control-PM: post mortem kidneys; control-TN: samples from tumor nephrectomies. Normalized pseudobulk expression values for MUC1 mRNA per cell type and individual were plotted as coloured data points on top of respective boxplot. Unpaired, two-sided Wilcoxon test, *P < 0.05, **P < 0.01.

In order to test for hypoxic regulation of *MUC1*, we exposed freshly isolated human renal tubular cells (PTC, n = 20 individuals) to 1 mM of the HIF-stabilizing agent dimethyl oxalylglycin (DMOG), hypoxia (1% O_2_) or control conditions (20.9% O_2_, no treatment). Compared to control, both stimuli increased MUC1 mRNA levels 3.5-fold (SD ± 1.77) or 2.4-fold (SD ± 0.95), respectively (Fig 1A). We confirmed increases of *MUC1* expression on protein level in immunoblot experiments in cells from two independent individuals (Fig 1B). To explore whether HIF directly regulates *MUC1* expression, we resorted to HIF ChIP-seq datasets from PTC (Grampp et al, 2016). We detected robust ChIP-seq signals for HIF-1α and its dimerization partner HIF-1β just upstream of the *MUC1* transcriptional start site (Fig 1C). To confirm HIF interactions with the promoter-proximal regulatory site of *MUC1* in a broader range of freshly isolated cells, we examined DNA-interactions of HIF-1α and HIF-1β by qRT-PCR in 17 additional HIF-ChIP experiments conducted under DMOG or untreated control conditions. This analysis revealed robust HIF recruitment to the *MUC1* promoter across a large selection of isolated PTC corroborating the findings from the HIF ChIP-seq experiments (Fig S1A). ChIP-qRT-PCR against a non-HIF-binding locus on chromosome 11 and the well-characterized *EGLN3* intronic enhancer was used as negative or positive control, respectively (Fig S1B and C) (Pescador et al, 2005). In line with a direct role of HIF in regulating *MUC1* in tubular cells, knocking-down HIF-1α or HIF-1β mRNA using siRNA abolished induction of MUC1 mRNA by DMOG in PTC (Fig 1D). *MUC1* expression has been predominantly detected in the TAL of the loop of Henle and more distal tubular segments of the human kidney (Heyderman et al, 1979; Knaup et al, 2018). To explore whether *HIF* and *MUC1* are co-expressed in these nephron segments, we re-analyzed available single-nucleus RNA-seq data covering a broad selection of human tissues (Han et al, 2020). Highest fractions of MUC1-positive cells were detected in stomach (40%), lung (30%), kidney (19%), transverse colon (18%), and pancreas (14%) (Fig S2A and Table S1). However, comparing the co-expression of MUC1 and HIF-1α mRNA revealed the highest degree of overlap of both transcripts in renal cells (Figs 1E and S2A and Table S1). Of note, these cells scored positive for mRNA expression of UMOD, SLC12A1, CALB1, and SLC4A1 indicating that MUC1 and HIF-1α mRNA expression overlaps in cells assigned to distal parts of the nephron. Similar results were obtained from published single-nucleus RNA-seq data derived from the kidney (Fig S2B and Table S2) (Zhang et al, 2021). In a recent snRNA-seq study, acutely injured human kidneys revealed a remarkable hypoxic transcriptional signature predominantly in tubular cells when compared with non-injured cells (Hinze et al, 2022). To test for a potential role of hypoxia in regulating *MUC1* expression in the distal tubule segments in cells suffering from acute kidney injury, we reanalyzed these single-cell RNA-seq data. In line with the results presented above and a role for hypoxia in regulating *MUC1*, MUC1 mRNA expression levels were significantly higher in distal tubular cells derived from the injured kidneys supporting a direct regulatory role for hypoxia in MUC1 expression in vivo (Fig S3). So far, our data suggest that HIF directly drives *MUC1* expression via a regulatory DNA element just upstream of the gene in renal tubular cells and especially in tubular segments that are likely to be involved in the development of *MUC1*-associated kidney diseases such as ADTKD.

Table S1. MUC1 and HIF-1α expressing cells in different human organs. Single-cell mRNA-seq data from all major adult human organs (data are from Han et al [2020]). Same data as in main Figs 1E and S2A. Overview of MUC1 and HIF-1α mRNA expressing cells in different human organs. Data are shown in absolute cell numbers and in % of cells positive for MUC1, HIF-1α or both transcripts.

Table S2. MUC1 and HIF-1α expressing cells in the kidney sorted according to marker genes for tubular origin. Single-cell RNA-seq data acquired in adult kidneys (raw data was published by Zhang et al [2021]). Same data as in Fig S2B. Overview of MUC1 and HIF-1α mRNA expressing cells in the kidney sorted according to marker genes for tubular origin. Data are shown in absolute cell numbers and in % of cells expressing the respective marker gene.

**Figure 2.**
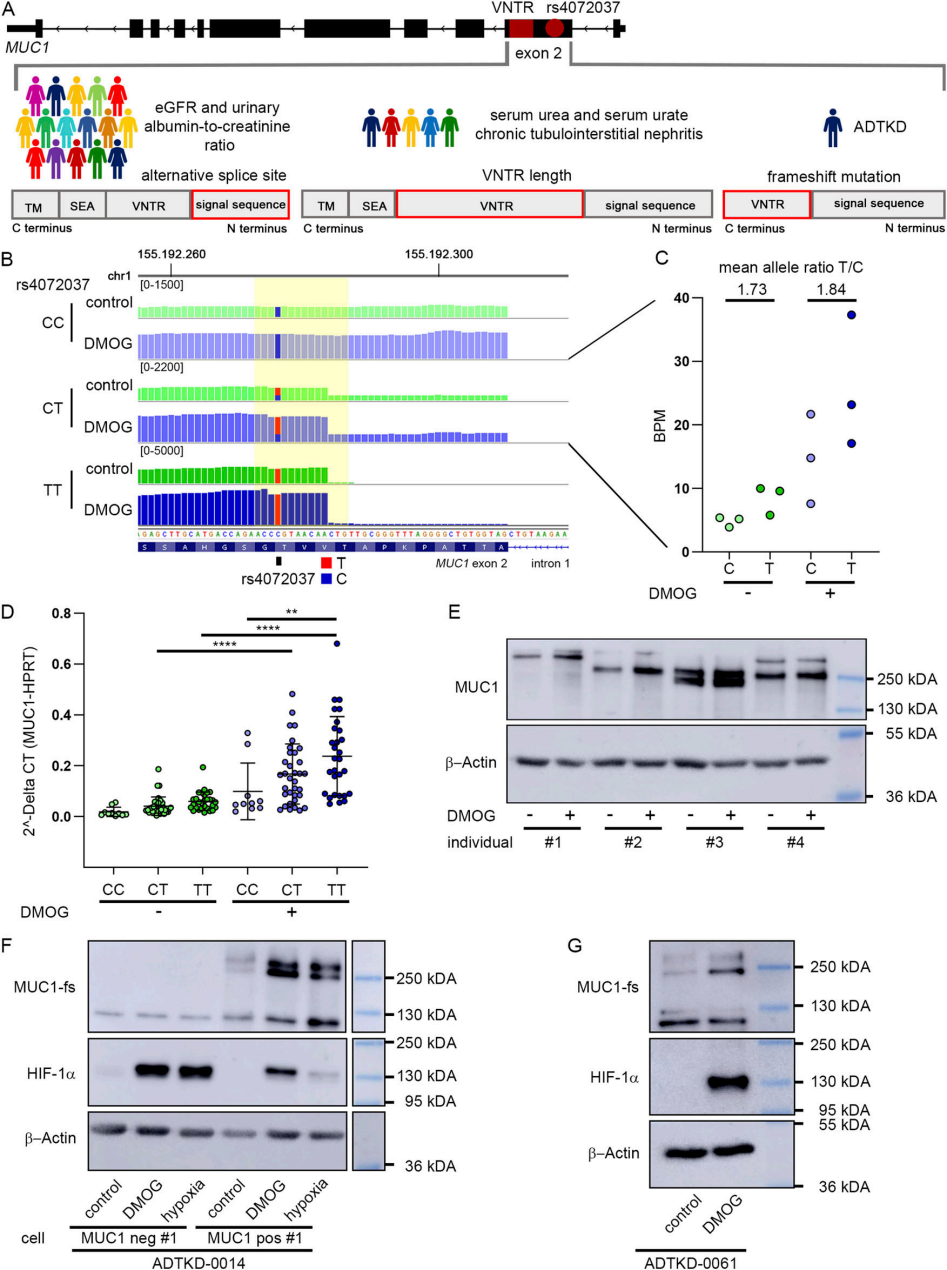
Stabilization of HIF increases expression of *MUC1* pathogenic variants. **(A)** Schematic overview of renal traits associated with genetic variation at the *MUC1* locus. The number of individuals schematically illustrates the size of the population affected by the variants. **(B)** RNA-seq tracks at the boundary of intron 1 and exon 2 at the *MUC1* locus from PTC of three individuals with different genotypes of the eGFR-associated SNP rs4072037 (CC, CT, TT). PTC were exposed to 1 mM DMOG for 16 h or left untreated. Of note, the T allele associates with a −27-nucleotide deletion transcript variant. Yellow box highlights the SNP rs4072037 and the alternative splice site created by the risk allele T of this SNP. Scaling indicates number of reads. **(B, C)** Ratios of normalized RNA-seq reads (bin per million, BPM) at the splice site for long transcripts carrying the C allele and alternatively spliced transcripts (T) were determined in untreated and DMOG-treated cells from three individuals heterozygous for rs4072037 including the individual from (B). **(D)** Expression qRT-PCR analysis of MUC1 mRNA in PTC exposed to 1 mM DMOG for 16 h or cultured under standard conditions. Samples were stratified according to the rs4072037 genotype (CC = 10, CT = 34, TT = 29). Values were normalized to expression levels of the housekeeping gene *HPRT*. Each dot represents the mean expression value from cells of one individual. qRT-PCR was performed in duplicates per individual. Bars indicate mean values ± SD. Two-way ANOVA followed by pairwise comparison **P < 0.01, ***P < 0.001. **(E)** Immunoblot for MUC1 and β-actin from lysates of PTC isolated from four different individuals. Cells were incubated with 1 mM DMOG for 16 h or left untreated. Please note that the size of the MUC1 protein varies intra- and inter-individually in dependence on the VNTR length of each allele. Densitometry measurements of MUC1 band intensity normalized for respective loading control β-actin reveals increased expression of MUC1 protein because of HIF stabilization (individual #1: 4.7-fold induction, individual #2: 2.9-fold induction, individual #3: 2.6-fold induction, individual #4: 1.3-fold induction). **(F)** Immunoblot for the frameshift variant of MUC1 (MUC1-fs), HIF-1α, and β-actin from lysates of human urine-derived primary tubular cells from ADTKD-*MUC1* patient ADTKD-0014. Two different cultures of cells were characterized according to the level of MUC1 expression as MUC1 negative (MUC1 neg #1) or MUC1 positive (MUC1 pos #1). Cells were exposed to 1 mM DMOG, 1% O_2_ (hypoxia) for 16 h or left untreated. **(G)** Immunoblot for the frameshift variant of MUC1 (MUC1-fs), HIF-1α, and β-actin from lysates of human urine-derived primary tubular cells donated by an additional patient affected by ADTKD-*MUC1* (ADTKD-0061) not related to individual ADTKD-0014. Cells were incubated with 1 mM DMOG for 16 h or left untreated. Source data are available for this figure.

Deleterious effects of *MUC1* expression on kidney function are caused by various genetic alterations including the very common polymorphism rs4072037, length of the VNTR, and rare variants in or close to the VNTR (Fig 2A) (Kirby et al, 2013; Kottgen et al, 2013; Ekici et al, 2014; Xu et al, 2018; Mukamel et al, 2021). Therefore, we wanted to explore whether HIF is able to transactivate these pathogenic variants in tubular cells.

Firstly, we generated RNA-seq data from PTC isolated from the kidneys of three individuals with different rs4072037 genotypes. Confirming the described effect of rs4072037 on alternative splicing of *MUC1*, we readily detected transcripts with the 27-nucleotide deletion caused by rs4072037 in RNA-sequencing reads covering the 5′ region of exon 2 of *MUC1* from individuals either heterozygous or homozygous for the T allele (Fig 2B). Similar to results from earlier studies on whole kidney lysates, the ratio of expression between the wild-type and the 27-nucleotide deletion variants was skewed to the shorter risk variant under basal conditions in cells from the three heterozygous individuals examined by RNA-seq (Fig 2C) (Xu et al, 2018). We observed increased absolute expression of total *MUC1* and the risk allele T in RNA-seq when cells were exposed to DMOG. However, the allele ratio of the mRNA remained unchanged under DMOG conditions (Fig 2C). We confirmed genotype-specific effects in tubular cells isolated from healthy parts of kidneys of a large cohort of 73 individuals undergoing tumor nephrectomy. The cells from these individuals were exposed to control conditions or DMOG to stabilize HIF. Expression of total MUC1 mRNA was quantified by qRT-PCR and samples were stratified according to the underlying genotype of rs4072037. Consistent with the data from the RNA-seq experiments and with whole kidney expression data from the recent study, we determined higher MUC1 mRNA levels in cells carrying the risk allele T under basal conditions (Fig 2D) (Xu et al, 2018). Although stabilizing HIF with DMOG increased expression of MUC1 across all genotypes, highest levels of MUC1 were determined in cells from TT individuals carrying the highest risk allele dosage (Fig 2D). Thus, our results indicate that HIF is an upstream regulator of *MUC1* that leads to induction of various MUC1 transcripts including the described splice variants associated with impaired kidney function.

**Figure S4.**
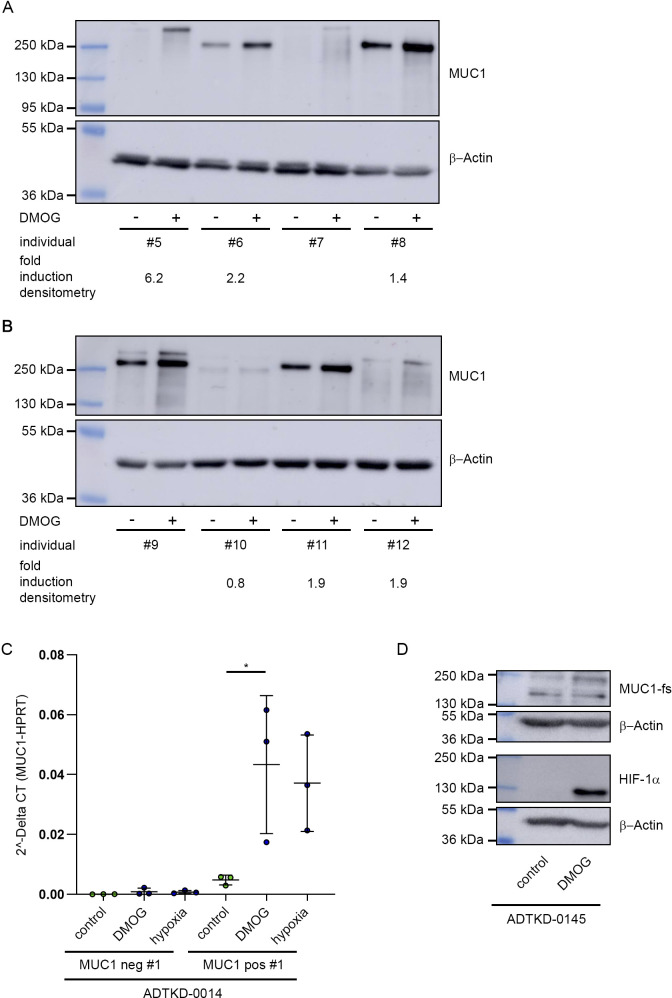
Stabilization of HIF promotes the expression of *MUC1* pathogenic variants. **(A, B)** Immunoblot for MUC1 and β-actin from lysates of primary tubular cells isolated from kidneys of eight additional individuals (please compare with main Fig 2E). Cells were incubated with 1 mM DMOG for 16 h or cultured under untreated standard conditions. Please note that the size of MUC1 protein varies with an intra- and interindividual different lengths of the VNTR. Fold-induction of MUC1 expression due to HIF stabilization was determined by densitometry. **(C)** MUC1 mRNA expression in human urine-derived tubular cells from patient ADTKD-0014 (same as in main Fig 2F) after incubation with 1 mM DMOG or hypoxia 1% O_2_ for 16 h as indicated. Bars are mean values ± SD from three independent experiments. Two-way ANOVA *P < 0.05. **(D)** Immunoblot for the frameshift variant of MUC1 (MUC1-fs), HIF-1α, and β-actin from lysates of human urine-derived primary tubular cells donated by a third patient affected by ADTKD-*MUC1* (ADTKD-D0145) not related to the individuals ADTKD-0014 and ADTKD-0061. Cells were treated with 1 mM DMOG for 16 h or left untreated. Source data are available for this figure.

Secondly, alleles with longer VNTR (>55 repeats) in exon 2 of *MUC1* have been linked to increased levels of serum urea und serum urate as biomarkers for reduced kidney function and with the risk for chronic tubulointerstitial nephritis as opposed to shorter VNTRs (Mukamel et al, 2021). We therefore screened protein lysates from primary renal tubular cells of 12 individuals exposed to HIF stabilizers for MUC1 proteins with different molecular weights. Although we cannot determine the exact length of the VNTR from the protein results for each individual, in 10 out of 12 samples, we observed robust HIF-mediated induction of variants with varying VNTR lengths resulting in different protein sizes ranging between ∼200–500 kD (Figs 2E and S4A and B) (Nath & Mukherjee, 2014). Thus, HIF drive the expression of *MUC1* including alleles with longer VNTR lengths, which are associated with biomarkers of reduced kidney function.

Thirdly, rare *MUC1* frameshift mutations cause the development of ADTKD-*MUC1* (Kirby et al, 2013; Ekici et al, 2014; Olinger et al, 2020). To explore whether HIF is capable of regulating the expression of these pathogenic variants, we isolated tubular cells from the urine (hUPTC) of three non-related ADTKD-*MUC1* patients. First, we used cells with high levels of *MUC1* expression and control cells with no *MUC1* expression from one patient (ADTKD-0014) for experiments. By exposing cells to DMOG or hypoxia (1% O_2_), we determined an increase of total MUC1 mRNA levels in the MUC1-positive cells when compared with untreated control cells (Fig S4C). Importantly, immunoblot analysis confirmed induction of the deleterious MUC1-fs protein upon HIF-stabilization in MUC1-positive cells (Fig 2F). We confirmed these results in MUC1-fs expressing cells isolated from the urine of two additional, non-related patients with ADTKD-*MUC1* (ADTKD-D0061 and ADTKD-0145; Figs 2G and S4D).

**Figure 3.**
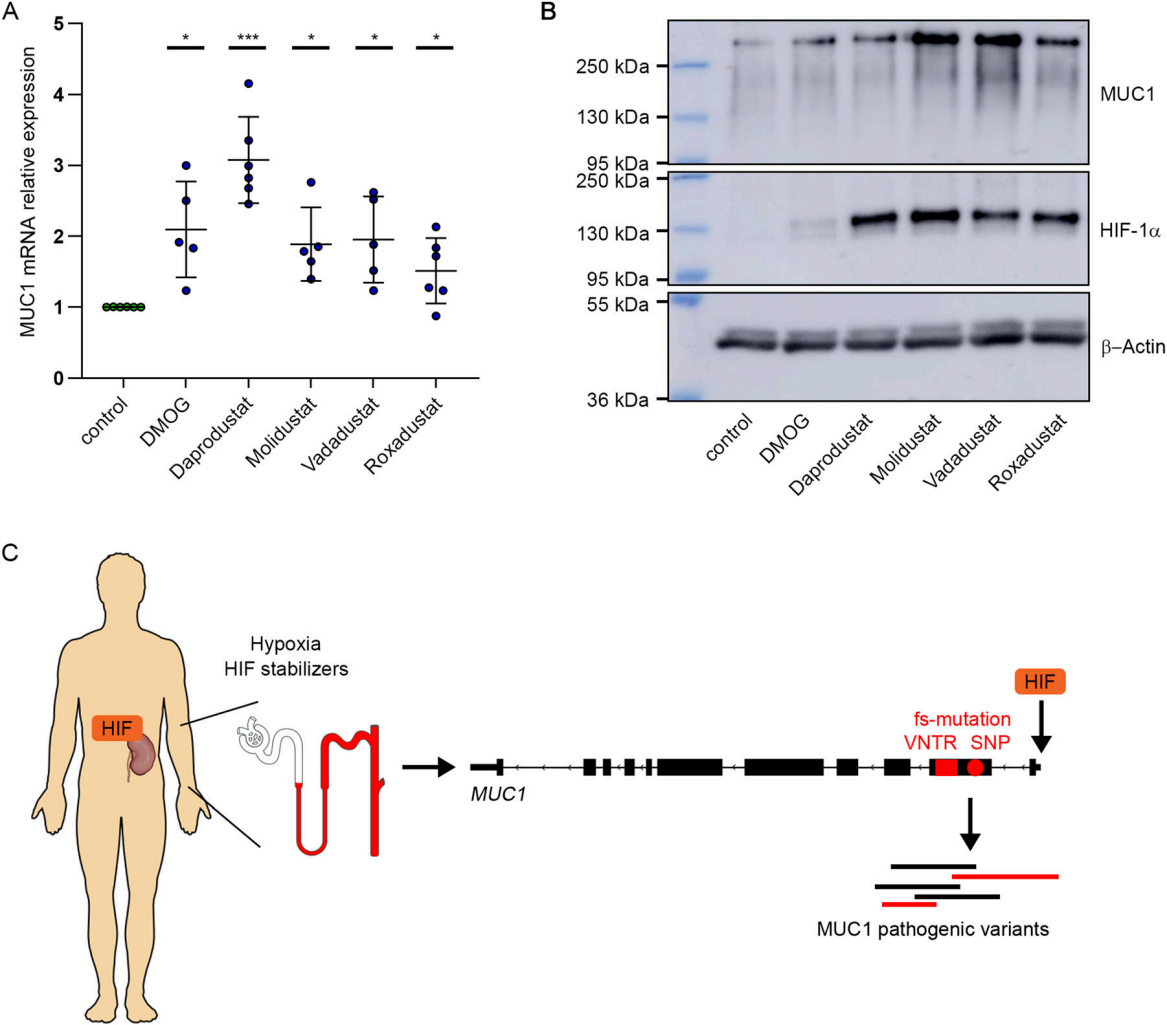
HIF-stabilizing compounds regulate *MUC1* expression. **(A)** MUC1 mRNA expression in PTC derived from five or six individuals was measured by qRT-PCR after pharmacological stabilization of HIF. Cells were treated with DMOG (1 mM), Daprodustat, Molidustat, Vadadustat or Roxadustat (each 100 μM) or control condition for 16 h as indicated. Bars indicate mean values ± SD. One sample *t* test of MUC1 mRNA expression levels in treatment conditions versus theoretical value of one representing no regulation, *P < 0.05, ***P < 0.001. **(A, B)** Immunoblot analyses of protein lysates generated under similar conditions as outlined in (A). Blots were exposed to anti-MUC1, anti-HIF-1α or anti β-actin antibodies. **(C)** Schematic view of potential effects of HIF stabilization on the expression of *MUC1* pathogenic variants. *MUC1* expressing more distal nephron segments are highlighted in red. Source data are available for this figure.

**Figure S5.**
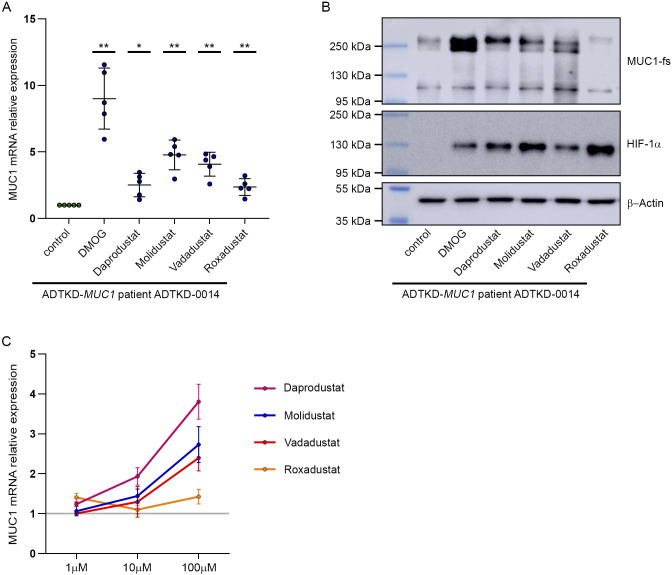
HIF-stabilizing compounds regulate *MUC1* expression. **(A)** MUC1 mRNA expression in urine-derived primary tubular cells (hUPTC) from patient ADTKD-0014 after incubation with DMOG (1 mM), Daprodustat, Molidustat, Vadadustat, Roxadustat (each 100 μM) or untreated control conditions for 16 h as indicated. Bars indicate mean values ± SD from five independent experiments. One sample *t* test of MUC1 mRNA expression levels in treatment conditions versus theoretical value of one representing no regulation, *P < 0.05, **P < 0.01. **(B)** Immunoblot for MUC1-fs, HIF-1α, and β-actin from lysates of hUPTC from ADTKD-*MUC1* patient ADTKD-0014. Cells were incubated with DMOG (1 mM), Daprodustat, Molidustat, Vadadustat, Roxadustat (each 100 μM) or control conditions for 16 h as indicated. **(C)** qRT-PCR analysis of MUC1 expression in primary tubular cells treated with different concentrations of HIF-stabilizing compounds for 16 h as indicated. Mean values of experiments with primary tubular cells from four different individuals are shown. Bars indicate mean values ± SEM. Source data are available for this figure.

HIF stabilizers have been approved for clinical use in several countries for treatment of the renal anemia. Therefore, we investigated the capability of a selection of these substances (Daprodustat, Molidustat, Vadadustat, Roxadustat) to induce *MUC1* expression in tubular cells. Strikingly, HIF stabilizers led to a robust induction of MUC1 mRNA and protein in a selection of primary cells of both *MUC1* wild-type and MUC1-fs individuals and at varying concentrations (Figs 3A and B and S5).

Taken together, our study links regulation of very common and very rare pathogenic variants of the kidney-disease gene *MUC1* with the HIF pathway in renal tubular cells, which is activated in acute and chronic kidney disease and upon treatment with anti-anemic drugs.

## Discussion

Genetic alterations comprising common low-effect to rare large-impact variants of kidney disease genes such as *UMOD* or *MUC1* determine the susceptibility to the development of CKD (Trudu et al, 2013; Olinger et al, 2020; Olinger et al, 2022). However, the broad heterogeneity observed in the course of CKD suggests a strong involvement of environmental factors in addition to genetic predisposition. With regard to the limited therapeutic options to treat CKD, successful preventive strategies depend on the identification of these environmental factors which may affect renal cellular function, for example, by transcriptional regulation of kidney disease genes.

In the monogenic kidney disease ADTKD-*MUC1*, effects of the deleterious *MUC1* variant are clearly restricted to a kidney phenotype (Devuyst et al, 2019). More specifically, our data confirm that expression of the frameshift variant MUC1-fs and alternative splicing as well as transcription of long VNTR alleles of *MUC1* do occur in renal epithelial cells. This indicates that the affected alleles operate to damage the kidney directly in the *MUC1*-expressing tubular segments and potentially the surrounding interstitium of the kidney. Our experiments show that HIF binding to a promoter-proximal element of *MUC1* increases expression of pathogenic *MUC1* variants (Fig 3C). The prerequisite for this regulation is given by the striking overlap of HIF-1α mRNA-positive and MUC1 mRNA-positive tubular cells in the single-nucleus RNA-seq datasets.

**Figure S6.**
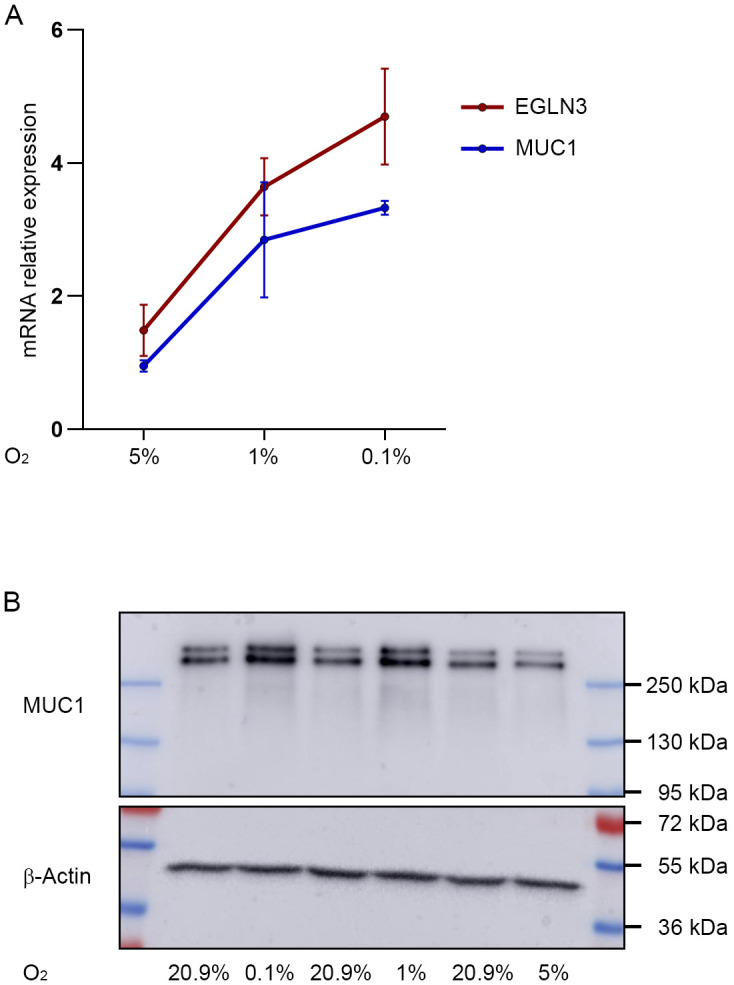
Severity of hypoxia influences *MUC1* expression. **(A)** MUC1 und EGLN3 mRNA expression in primary tubular cells exposed to different levels of hypoxia for 16 h as indicated. Mean values ± SD of experiments using PTC from three different individuals are shown. Values were normalized to expression levels of the housekeeping gene *HPRT* and to values from cells kept under control conditions. **(B)** Representative immunoblot for MUC1 and β-actin from lysates of PTC of one donor incubated for 16 h under hypoxic or control conditions (O_2_ 20.9%) as indicated. Source data are available for this figure.

HIF stabilization and subsequent increases in *MUC1* expression in renal tubular cells may occur because of reduced oxygen supply or a hypoxic microenvironment in the context of many renal diseases such as acute kidney injury or CKD and renal inflammation (Howie, 1986; Haase, 2013; Nangaku et al, 2013; Schodel & Ratcliffe, 2019; Hinze et al, 2022). Of note, we observed that MUC1 mRNA and protein increased in PTC with lowering of oxygen concentration in the hypoxic chamber from 5% to 0.1% (Fig S6). This indicates that *MUC1* regulation by HIF is sensitive to very low oxygen levels. In line with this hypothesis, recent single-cell RNA-sequencing data have shown MUC1 mRNA to be up-regulated in tubular cells in acutely injured human kidneys, which display a striking hypoxic transcriptional signature (Hinze et al, 2022). Although *MUC1* expression and induction were most prominent in distal tubule segments, MUC1 mRNA was also increased significantly in proximal tubule cells (Fig S3). This finding is in keeping with a recent study detecting elevated *MUC1* levels in proximal tubule cells in kidneys from mice and humans after ischemic damage (Al-Bataineh et al, 2021). In the setting of experimental acute kidney injury, a protective role of MUC1 has been identified in the early phase of the insult in mouse models of ischemia reperfusion injury (Pastor-Soler et al, 2015; Gibier et al, 2017). However, in one study, *Muc1* deletion led to reduced kidney fibrosis in later phases of the injury indicating a pro-fibrotic potential of wild-type *Muc1* in the mouse kidney (Gibier et al, 2017). Apart from the possibly pro-fibrotic potential of wild-type MUC1, growing evidence supports harmful effects of genetic variants at the *MUC1* locus on kidney function (Xu et al, 2018; Olinger et al, 2020; Mukamel et al, 2021).

In patients carrying *MUC1* risk variants, recurrent episodes of acute kidney injury or chronic states of inflammation leading to HIF stabilization could potentially accelerate the development of CKD in the long run by increasing the abundance of toxic protein variants (Schodel & Ratcliffe, 2019). In this respect, in ADTKD-*UMOD* patients with homozygous mutations, the loss of eGFR was accelerated when compared with heterozygous family members (Rezende-Lima et al, 2004; Edwards et al, 2017). This observation would support a dose-dependent effect of the deleterious proteins in ATDKD proteinopathies and HIF stabilization may contribute to higher levels of MUC1-fs.

Many patients with reduced kidney function will require the start of an anti-anemia therapy at some point during the course of the disease. Interestingly, patients from the UK Biobank cohort with longer *MUC1* VNTR alleles also displayed lower hemoglobin levels possibly reflecting reduced kidney function (Mukamel et al, 2021). This will inevitably lead to therapeutic measures to treat anemia in some of these individuals. Because HIF stabilizers have been licensed for the treatment of the renal anemia in non-dialysis-dependent patients in many countries, we suspect that individuals with *MUC1* risk variants will be frequently exposed to this treatment even before starting renal replacement therapy. Our data create the hypothesis that in patients carrying risk alleles of the *MUC1* gene, HIF stabilizers could lead to increased levels of the harmful protein and further reduce kidney function by accelerating the disease. Though HIF stabilizers have been designed to increase EPO production in interstitial cells via induction of HIF-2α, they do not selectively stabilize one of the two HIF isoforms (Yeh et al, 2017). In rodents, treatment with HIF stabilizers led to robust stabilization of HIF-1α in tubular segments and, in the setting of acute kidney injury, preconditional HIF activation appears to be protective (Bernhardt et al, 2006; 2009; Schley et al, 2012; Ito et al, 2020). Furthermore, the pleiotropic effects observed with some of the compounds, for example, on iron and cholesterol homeostasis imply that cell types of other than the interstitial origin in the kidney can stabilize HIF upon treatment with HIF stabilizers and that this will lead to a broader HIF response in the body (Haase, 2017). In this respect, the HIF pathway has been implicated in promoting interstitial fibrosis and cyst growth in models of CKD and autosomal-dominant polycystic kidney disease, respectively (Kimura et al, 2008; Kraus et al, 2018). Based on these and our findings, it will be of great interest to see whether other potentially harmful pathways in the kidney become active in patients receiving long-term HIF-stabilizing therapy.

In conclusion, this study highlights the interplay between genetic predisposition and environmental factors potentially influencing the course of kidney disease by modulating the expression of harmful variants of *MUC1*. Based on our findings, we suggest that ADTKD-*MUC1* patients not yet requiring dialysis should be treated with great caution when using HIF-stabilizing compounds as anti-anemic agents until there are more data available.

## Materials and Methods

### Patient-derived tubular cells

We isolated primary tubular cells from non-diseased tissue of tumor nephrectomy specimens from 73 donors and from the urine of three non-related patients affected by ADTKD-*MUC1* through *MUC1* VNTR mutations. *MUC1* mutations occurring in individuals ADTKD-0061 (family A-14) and ADTKD-0014 (family A-11) have been characterized by Knaup et al in previous work (Ekici et al, 2014; Knaup et al, 2018). The third ADTKD-*MUC1* patient is a direct descendent of the individual ADTKD-0145 (family A-30) published by Wenzel et al, in whom the mutation has been confirmed (Wenzel et al, 2018). Cells were cultured according to established protocols (Keller et al, 2012; Wiesener et al, 2020). Tubular cells were exposed to HIF stabilizers (concentrations used were: DMOG 1 mM, Daprodustat 100 μM, Molidustat 100 μM, Vadadustat 100 μM, Roxadustat 100 μM or as indicated), hypoxia (1% O_2_ or control conditions [20.9% O_2_, no treatment]) for 16 h.

### Chemicals

HIF stabilizers were purchased from Cayman Chemical Company (Cay71210; DMOG, Cay19915; Daprodustat, Cay15297; Molidustat = BAY 85-3934, Cay15295; Vadadustat, Cay15294; Roxadustat = FG-4592).

### RNA isolation, qRT-PCR, and siRNA experiments

We used lysates from tubular cells to isolate RNA as previously described (Lauer et al, 2020). Quantitative RT–PCRs were performed using Maxima SYBR Green/ROX qRT-PCR Master Mix (Thermo Fisher Scientific) on a StepOnePlus Real-Time PCR cycler (Applied Biosystems). Primers are listed in Table S3. siRNA experiments against HIF were conducted as described by Lauer et al (2020).

Table S3. Oligonucleotides used in this study.

### Western blotting

MUC1 protein was detected by immunoblotting using a monoclonal anti-MUC1 antibody (D9O8K, rabbit monoclonal, dilution: 1:1,000; Cell Signaling Technology). MUC1 frameshift (MUC1-fs) protein was detected using a polyclonal anti-frameshift MUC1 antibody (rabbit polyclonal, dilution 1:5,000; Pineda Antibody Service) as described earlier (Knaup et al, 2018). HIF-1α was detected applying a polyclonal anti-HIF-1α antibody (Cay10006421, rabbit polyclonal, dilution: 1:1,000; Cayman Chemicals). Specificity of the anti-HIF-1a antibody was demonstrated in previous work (Lauer et al, 2020; Protze et al, 2022). A horseradish peroxidase-conjugated anti-rabbit antibody served as secondary antibody (P0399, swine polyclonal, dilution: 1:2,500; Agilent Technologies). β-actin was detected by using a monoclonal anti–β-actin-peroxidase antibody (A3854, mouse monoclonal, dilution: 1:60,000; Sigma-Aldrich). Band intensity of MUC1 and β-actin protein was quantified by densitometry using ImageJ2 software (version 2.35) (National Institutes of Health).

### Genotyping

We genotyped genomic DNA isolated from primary tubular cells using the Infinium Global Screening Array-24+ v3.0 (Illumina). Data were analyzed with the GenomeStudio software (v.2.0.5; Illumina) using the Infinium Global Sreening Array v3.0 Manifest file (BPM Format GrCh37).

### RNA-sequencing

We performed RNA-seq on isolated RNA from lysates of tissue-derived primary renal tubular cells exposed to 1 mM DMOG or control conditions for 16 h. Libraries were prepared using the Illumina protocol and sequenced on a HiSeq 2500 (Illumina). 100 bp single-end sequencing was performed on RNA samples from primary tubular cells yielding ∼30 million reads per sample. After quality check using FastQC (v0.11.8), reads were aligned to the human reference genome (hg38) using the STAR alignment software (v2.6.1c) (Dobin et al, 2013). Quality metrics for RNA-seq data are listed in Table S4. Sorted BAM files of primary renal tubular cells exposed to 1 mM DMOG or control conditions were converted to BigWig (BW) files using the deepTools function of deepTools (v3.5.0) (Ramírez et al, 2016). Normalization was performed using the parameter bins per million mapped reads (BPM) with a bin size of 1 bp. BPM values + and − 1 bp from the MUC1 intron 1 exon 2 splice site were determined by using the Integrative Genomics Viewer (version 2.8.4).

Table S4. RNA-seq quality metrics. Table shows mapped reads and RNA integrity number for respective RNA-seq samples.

### Chromatin immunoprecipitation

ChIP experiments in PTC were performed essentially as described (Grampp et al, 2016; Grampp et al, 2017). Two 15 cm dishes of sub-confluent PTC were used and exposed to 1 mM DMOG or control conditions for 16 h. Sonification was carried out with a Bioruptor Plus sonicator (Diagenode) using 28 cycles with 15 s on and 15 s off. For immunoprecipitations, 6–10 μl of antibodies against HIF-1α (rabbit polyclonal, Cayman Chemicals; Cay10006421) or HIF-1β (rabbit polyclonal, Novus Biologicals; NB100-110) or control IgG (normal rabbit IgG, 12-370; Merck) were used. ChIP qRT-PCRs were performed using SYBR Green chemistry (Thermo Fisher Scientific) on a StepOnePlus real-time PCR cycler (Applied Biosystems). Primers are listed in Table S3. HIF ChIP-seq data have been published previously (Grampp et al, 2016; 2017).

### Single-cell RNA-sequencing data analysis

We accessed published Microwell-seq data at https://cellxgene.cziscience.com/e/human_cell_landscape.cxg/ (access: 1 Feb 2022) (Han et al, 2020). Cells were filtered for expression of MUC1 and HIF-1α mRNA using default settings of the CellXGene browser. Markers for different tubular segments were identified according to Zhang et al (2021). Kidney scRNA-seq data were downloaded as read count matrices from GEO (GSE159115), selected for benign kidney samples according to the published sample list and processed according to the specifications stated by Zhang et al using R (version 4.1.0) (Zhang et al, 2021). In short, low-quality cells (mitochondrial content >80%, <300 genes detected/cell) were discarded and Scrublet (Python package version 0.2.3, imported via R package reticulate version 1.24) was used to remove duplicates in each sample (Wolock et al, 2019). Genes detected in less than five cells and mitochondrial, ribosomal, and sex genes were removed. Gene annotations were retrieved via biomRt version 2.48.3 (Durinck et al, 2009). The total number of UMI was standardized to 5,000 per cell and log-transformed (ln[x+1]) using the NormalizeData function in Seurat (version 4.1.0) (Hao et al, 2021). Calling the fastMNN implementation in SeuratWrappers (https://github.com/satijalab/seurat-wrappers, access: 4 Feb 2022) batch correction was performed on 2,000 highly variable genes (HGV) (Haghverdi et al, 2018). Further downstream analysis in Seurat included computation and scaling of HVG, principal component analysis, and a 2-dimensional t-distributed stochastic neighbor embedding (*t*-SNE) based on the first 50 principal components. Cells were denoted “positive” if ≥ 1 raw UMI read(s) of the respective gene was counted.

snRNA-seq data of control and acute kidney injury (AKI) kidneys published by Hinze et al can be accessed as cellranger-mapped count data and metadata including cell-type assignments (Hinze et al, 2022). Data were downloaded from GEO (GSE210622) and processed with R package Seurat (version 4.1.0). Mapped count datasets were transformed into Seurat objects via functions “Read10X()” and “CreateSeuratObject(),” respectively. All objects were merged into one Seurat object, which was subset to the nuclei listed in the metadata table. Only nuclei that passed the quality control steps as described in Hinze et al (i.e., exclusion of nuclei with more than 5% mitochondrial reads or less than 500 detected genes and doublets) were kept, yielding a total number of 106,971 nuclei (Hinze et al, 2022). In addition, the merged Seurat object was filtered for genes expressed in ≥500 of all remaining nuclei. Using normalized counts only, our analysis did not require any further integration method or batch correction. For each cell type and individual, a pseudo bulk object was computed on raw counts via “AggregateExpression()” and then normalized to Counts per Million (CPM) via “NormalizeData()” with parameter choices “scale.factor = 1 × 10^6^” and “normalization.method = “RC”.” For each pseudobulk object, differential gene expression analysis was performed on raw counts with R package DESeq2 (version 1.38.2) as described in Hinze et al (2022) (i.e., for each cell-type condition, control was tested against condition AKI). The normalized pseudobulk expression of MUC1 RNA per cell type and individual was plotted as datapoint on top of respective boxplots. An unpaired, two-sided Wilcoxon test was performed via function “stat_compare_means (method = “wilcox.test”)” from R package ggprubr (version 0.5.0).

### Visualization

Adobe Photoshop Elements (Adobe Inc.) and Microsoft PowerPoint 2019 (Microsoft Corporation) were used for creating figures. Parts of Fig 3C were designed by using pictures from Servier Medical Art. Servier Medical Art by Servier is licensed under a Creative Commons Attribution 3.0 Unported License (https://creativecommons.org/licenses/by/3.0/).

### Statistics

Statistical analyses for RNA expression as determined by qRT-PCR or DNA enrichment as quantified by ChIP-qRT-PCR were performed applying the one-sample *t* test comparing the mean value with a hypothetical value of one or one- and two-way ANOVA analysis if applicable using GraphPadPrism Version 9.0.2 (GraphPad Software Inc.). Statistical analysis of expression values determined in RNA pseudobulk analysis was done by applying an unpaired, two-sided Wilcoxon test using R ggprubr (version 0.5.0) via function “stat_compare_means (method = “wilcox.test”).”

### Study approval

The local ethics committee at the University of Erlangen–Nürnberg approved the use of the tissue and cells (3755, 271_Bc, 251_18B). Each patient gave informed consent. Specimens were collected in accordance with the World Medical Association Declaration of Helsinki.

## Data Availability

HIF ChIP-seq data are available at GEO (GSE101064) and RNA-seq data are available at GEO (GSE224177). Kidney scRNA-seq data were downloaded from GEO (GSE159115) (Zhang et al, 2021). Single-nuclei RNA-sequencing data of control and AKI kidneys were downloaded from Hinze et al (2022) (GSE210622). Microwell-seq data published by Han et al (2020) can be accessed via https://cellxgene.cziscience.com/e/human_cell_landscape.cxg/ (access: 1 Feb 2022).

## Supplementary Material

Reviewer comments
