# Supplementary material for: Hypoxia controls expression of kidney-pathogenic MUC1 variants
Source: Life Sci Alliance. 2023 Jun 14;6(9):e202302078. doi: 10.26508/lsa.202302078 (PMC10267510; doi:10.26508/lsa.202302078)

Hypoxia controls expression of kidney-pathogenic *MUC1* variants

Stephanie Naas[1], René Krüger[1], Karl Xaver Knaup[1], Julia Naas[2], Steffen Grampp[1], Mario Schiffer[1], Michael Wiesener[1], Johannes Schödel[1]

Uncropped immunoblots

Figure 1 b

Muc1

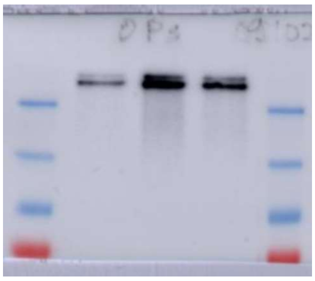

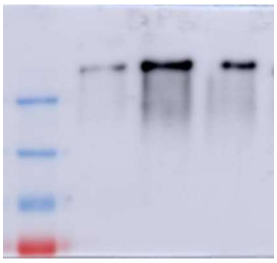

Hif-1a

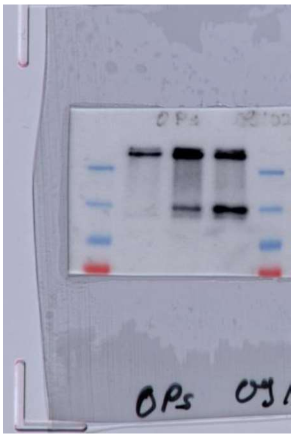

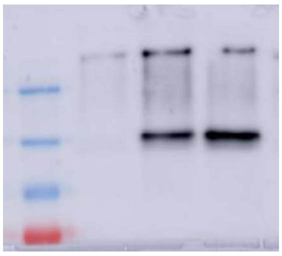

Actin B

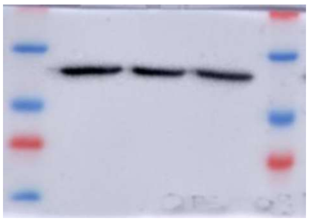

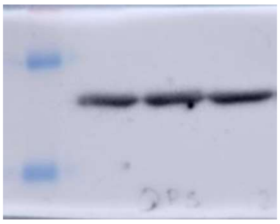

Figure 2

e

Muc1

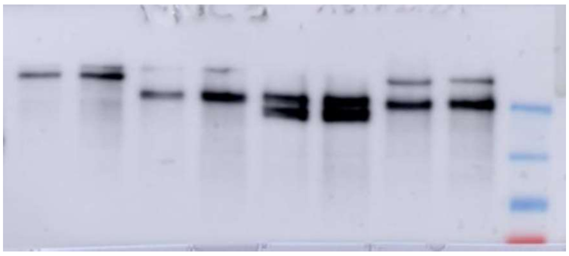

ActinB

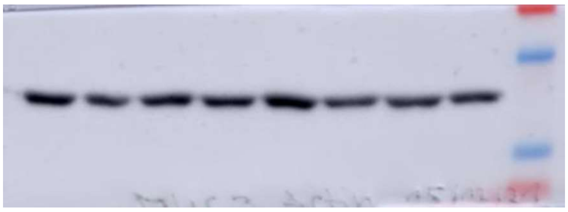

f

Muc FS

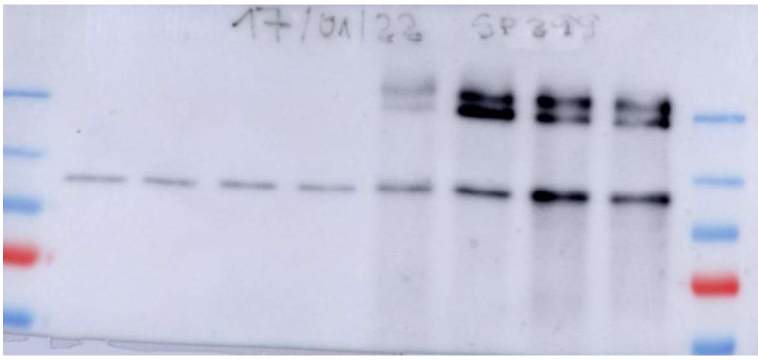

Hif-1a

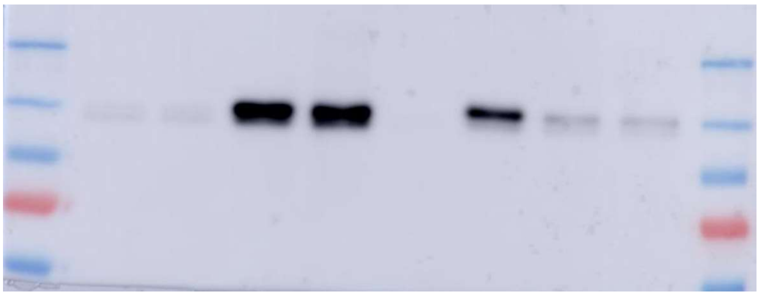

ActinB

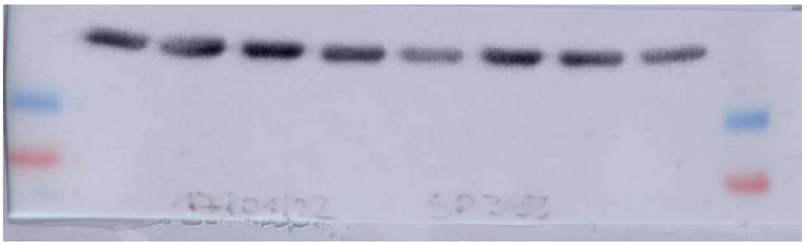

g

MUC1 fs

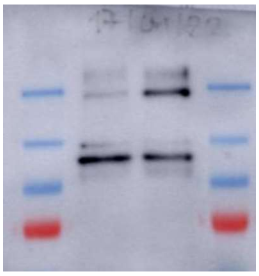

Hif1a

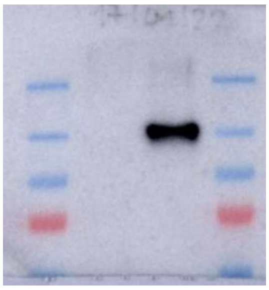

ActinB

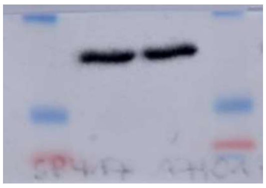

Figure 3

b

MUC1

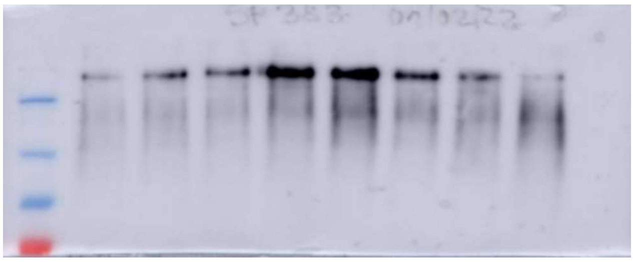

Hif1a:

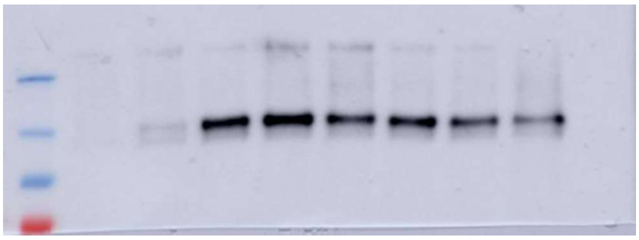

Actin:

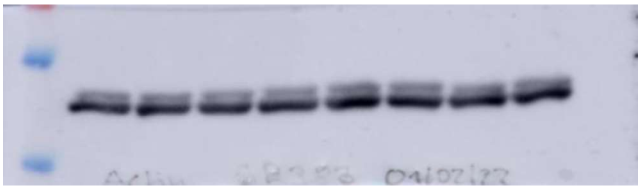

Supplemental Figure S4

a

MUC1

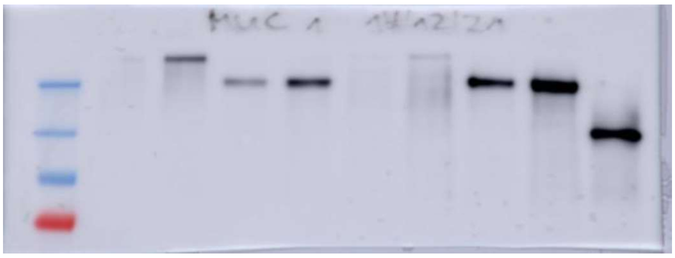

ActinB

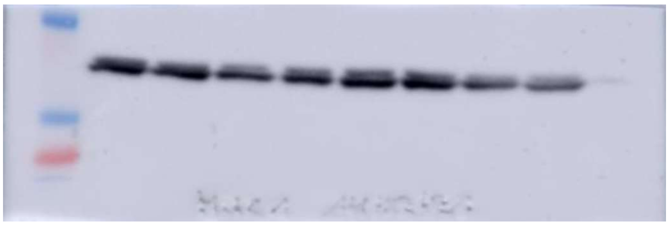

b

MUC1

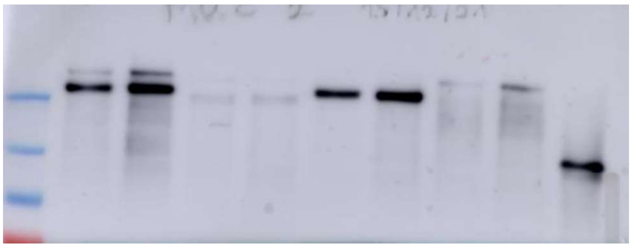

ActinB

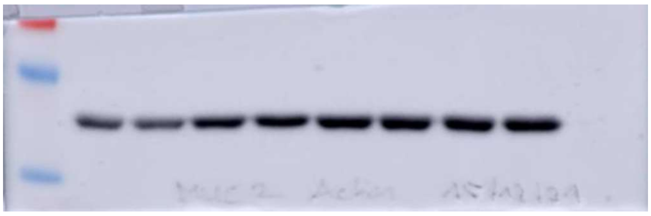

d

MUC1-fs

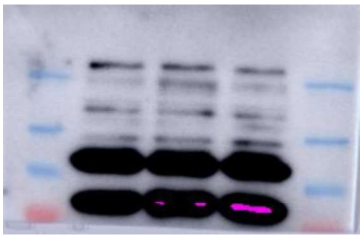

HIF-1a

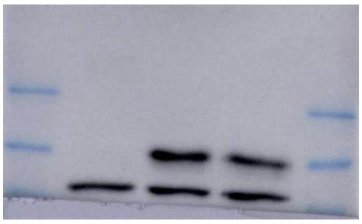

ActinB

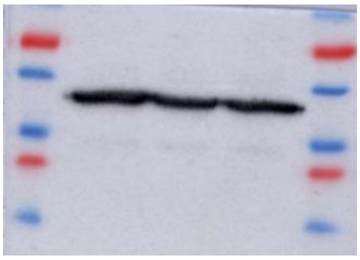

Supplemental Figure S5

b

MUC1 fs

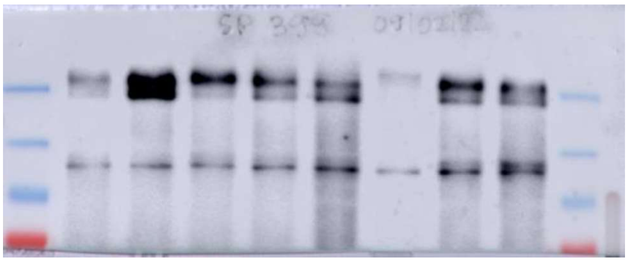

Hif-1a

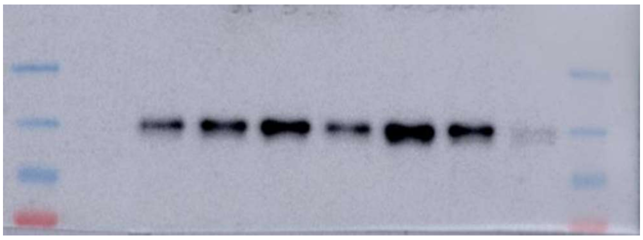

ActinB

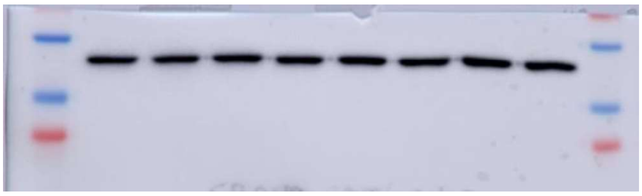

Supplemental Figure S6

b

MUC1

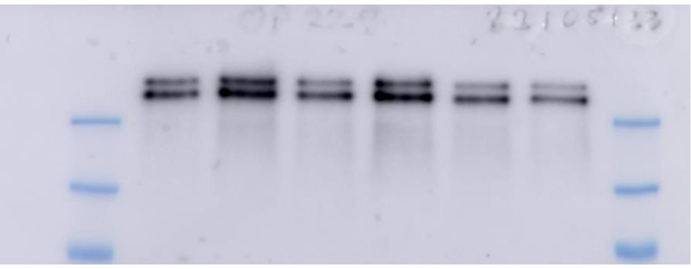

ActinB

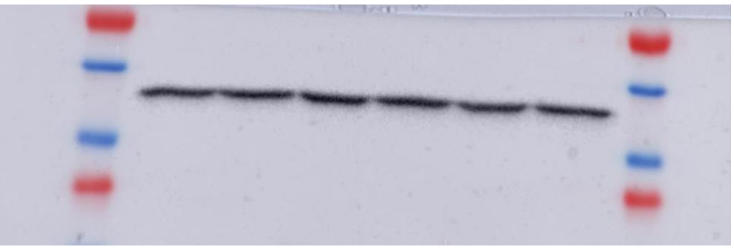

Supplement: Supplementary file 1 [file LSA-2023-02078_SdataF1_F2_F3_FS4_FS5_FS6.pdf]
