# Supplementary material for: Hypoxia controls expression of kidney-pathogenic MUC1 variants
Source: Life Sci Alliance. 2023 Jun 14;6(9):e202302078. doi: 10.26508/lsa.202302078 (PMC10267510; doi:10.26508/lsa.202302078)
Supplement: Supplementary file 4 [file LSA-2023-02078_TableS3.docx]

Supplementary Table 3

| **Expression primer** | **Sequence** |
| --- | --- |
| HPRT fw | GACCAGTCAACAGGGGACAT |
| HPRT rev | AACACTTCGTGGGGTCCTTTTC |
| MUC1 fw | TCCTTTCTCTGCCCAGTCTG |
| MUC1 rev | CAGCTGCCCGTAGTTCTTTC |
| **ChIP primer** | **Sequence** |
| MUC1_HIF_bind_fw | CTTCTAACCGCTCCCTGTCC |
| MUC1_HIF_bind_rev | TTCATCGGAGCCCAGGTTTA |
| EGLN3_positive_control_fw | AGTGTCCGTTCCCAGCTCAG |
| EGLN3_positive_control_rev | TAGGCACAGTAAACAGGCC |
| Chr11_negative_control_fw | GGATCACCAGGTGTATTCGG |
| Chr11_negative_control_rev | CATCCAAGGGGGATACACAC |
