# Supplementary material for: Hypoxia controls expression of kidney-pathogenic MUC1 variants
Source: Life Sci Alliance. 2023 Jun 14;6(9):e202302078. doi: 10.26508/lsa.202302078 (PMC10267510; doi:10.26508/lsa.202302078)
Supplement: Supplementary file 5 [file LSA-2023-02078_TableS4.docx]

Supplementary Table 4

| **Sample** | **Mapped reads** | **RIN Score** |
| --- | --- | --- |
| RNA_1_ctrl | 66415876 | 10 |
| RNA_1_DMOG | 57977035 | 10 |
| RNA_2_ctrl | 70852694 | 10 |
| RNA_2_DMOG | 54794777 | 9.5 |
| RNA_3_ctrl | 64406178 | 9.9 |
| RNA_3_DMOG | 67701393 | 9.9 |
| RNA_4_ctrl | 37529648 | 9.9 |
| RNA_4_DMOG | 38118739 | 10 |
| RNA_5_ctrl | 28000975 | 10 |
| RNA_5_DMOG | 38761121 | 10 |
