# Supplementary material for: Hypoxia controls expression of kidney-pathogenic MUC1 variants
Source: Life Sci Alliance. 2023 Jun 14;6(9):e202302078. doi: 10.26508/lsa.202302078 (PMC10267510; doi:10.26508/lsa.202302078)
Supplement: Reviewer comments [file LSA-2023-02078_review_history.pdf]

Life Science Alliance

Hypoxia controls expression of kidney-pathogenic MUC1 variants

Stephanie Naas, René Krüger, Karl Knaup, Julia Naas, Steffen Grampp, Mario Schiffer, Michael Wiesener, and Johannes Schödel
DOI: https://doi.org/10.26508/lsa.202302078

Corresponding author(s): Johannes Schödel, Universitätsklinikum Erlangen

Review Timeline:

Submission Date:	2023-04-05
Editorial Decision:	2023-05-05
Revision Received:	2023-05-24
Editorial Decision:	2023-05-31
Revision Received:	2023-05-31
Accepted:	2023-06-01

Scientific Editor: Novella Guidi

Transaction Report:

(Note: With the exception of the correction of typographical or spelling errors that could be a source of ambiguity, letters and reports are not edited. The original formatting of letters and referee reports may not be reflected in this compilation.)

May 5, 2023

Re: Life Science Alliance manuscript #LSA-2023-02078-T

Dr. Johannes Schödel
Uniklinikum Erlangen
Department of Nephrology and Hypertension
Friedrich-Alexander-University Erlangen-Nuremberg
Ulmenweg 18
Erlangen D-91054
Germany

Dear Dr. Schödel,

Thank you for submitting your manuscript entitled "Hypoxia controls expression of kidney-pathogenic MUC1 variants" to Life Science Alliance. The manuscript was assessed by expert reviewers, whose comments are appended to this letter. We invite you to submit a revised manuscript addressing the Reviewer comments.

To upload the revised version of your manuscript, please log in to your account: https://lsa.msubmit.net/cgi-bin/main.plex

You will be guided to complete the submission of your revised manuscript and to fill in all necessary information. Please get in touch in case you do not know or remember your login name.

While you are revising your manuscript, please also attend to the below editorial points to help expedite the publication of your manuscript. Please direct any editorial questions to the journal office.

The typical timeframe for revisions is three months. Please note that papers are generally considered through only one revision cycle, so strong support from the referees on the revised version is needed for acceptance.

When submitting the revision, please include a letter addressing the reviewers' comments point by point.

We hope that the comments below will prove constructive as your work progresses.

Thank you for this interesting contribution to Life Science Alliance. We are looking forward to receiving your revised manuscript.

Sincerely,

Novella Guidi, PhD
Scientific Editor
Life Science Alliance

A. THESE ITEMS ARE REQUIRED FOR REVISIONS

-- A letter addressing the reviewers' comments point by point.

-- An editable version of the final text (.DOC or .DOCX) is needed for copyediting (no PDFs).

-- High-resolution figure, supplementary figure and video files uploaded as individual files: See our detailed guidelines for preparing your production-ready images, https://www.life-science-alliance.org/authors

-- Summary blurb (enter in submission system): A short text summarizing in a single sentence the study (max. 200 characters including spaces). This text is used in conjunction with the titles of papers, hence should be informative and complementary to the title and running title. It should describe the context and significance of the findings for a general readership; it should be written in the present tense and refer to the work in the third person. Author names should not be mentioned.

-- By submitting a revision, you attest that you are aware of our payment policies found here: https://www.life-science-alliance.org/copyright-license-fee

B. MANUSCRIPT ORGANIZATION AND FORMATTING:

Full guidelines are available on our Instructions for Authors page, https://www.life-science-alliance.org/authors

We encourage our authors to provide original source data, particularly uncropped/-processed electrophoretic blots and spreadsheets for the main figures of the manuscript. If you would like to add source data, we would welcome one PDF/Excel-file per figure for this information. These files will be linked online as supplementary "Source Data" files.

IMPORTANT: It is Life Science Alliance policy that if requested, original data images must be made available. Failure to provide original images upon request will result in unavoidable delays in publication. Please ensure that you have access to all original microscopy and blot data images before submitting your revision.

Reviewer #1 (Comments to the Authors (Required)):

Naas and colleagues provide an interesting report on the role of hypoxic expression of the kidney pathogenic gene MUC1. The authors used patients' derived primary tubular cells to study the role of hypoxia and hypoxia factor-1 stabilizers to study MUC1 expression. They report that both hypoxia and prolyl hydroxylases inhibitors increased MUC1 expression in a HIF-dependent way. Moreover, the gene risk variant associated with the development of chronic kidney disease was increasingly expressed under prolyl hydroxylase inhibitor treatment.

As pointed out above the study is interesting and well written. However, a few questions I would like the authors to address.

1. The authors compared incubation at 20.9 (control - normoxia?) and hypoxia on 1% O2. Certainly, 20.9 % O2 is not a physiological oxygen concentration. This would be rather closer to the 1 % than to the 20.9. Do the authors have any data at intermediate oxygen concentrations, e.g. 5 - 6 % O2? This may be relevant, particularly in view of Fig. 1 E, where they find MUC1 expression in their single cell analyses in the human kidney.

2. In most of their experiments, the effect of hypoxia was weaker than treatment with DMOG (PAD inhibitor). Again, the authors have experience with oxygen concentrations below 1 %? Moreover, Fig. 1 B show samples derived from two individuals. For both western blots comparing MUC1 and HIF-1 expression, it appears as if MUC1 expression is higher with moderately lower HIF-1α levels. Please, comment.

3. Fig. 1 D with siRNA against HIF-1β indicates that there may be effects of DMOG other than through HIF signalling. With respect to HIF-1α siRNA, HIF-2α could be involved. In the discussion, the authors argue that PHD inhibitors are not selective. However, Daprodustat, one of the drugs to be used for HIF-2-dependent EPO expression, shows the strongest effect with respect to MUC expression. Do the authors have data with siRNA for HIF-1α and Daprodustat treatment?

Minor point:
Fig. 2 is cut off at the end.

Reviewer #2 (Comments to the Authors (Required)):

This manuscript highlighted the potential contribution of environmental factor on the course of kidney disease by modulating the expression of MUC1. The authors showed that stabilizing HIF-1a using different approaches induced MUC1 expression including the disease associated variants. Intriguingly, pharmacological compounds to stimulate EPO production by stabilizing HIF (MUC1 upstream regulator) in the diseased kidney of CKD patients before and during dialysis have been clinically approved. Thus, this study forewarns that using HIF stabilizing agents should be used with great caution in ADTKD MUC1 patients as these drugs may aggravate kidney disease by enhancing MUC1 expression including the deleterious variants.

1.MUC1 has been shown to be induced and play a key role in both human and mouse kidney proximal tubule cells following ischemia reperfusion injury (PMID: 26739894 and 34151589). The authors should consider in their introduction and discussion sections the role of MUC1 in proximal tubule where damage is most significant following the ischemia (hypoxia) injury protocol.

2.Used anti-HIF-1a antibody (Cay1000062421) in the study needs to be validated as the authors considered the 130 kDa band represents HIF-1a, while the antibody's company website considered the 130 kDa band as nonspecific band and the corrected band size for HIF-1a is ~100 kDa. One blot was cut just below 130 kDa and it's hard to see if there is any band ~100 kDa.

3.Statistical analysis needs to be revised again as t-test can be used to compare two different groups only, while for three or more groups (the case of most figures) ANOVA need to be performed followed by pairwise comparison.

Hypoxia controls expression of kidney-pathogenic MUC1 variants

Stephanie Naas, René Krüger, Karl Xaver Knaup, Julia Naas, Steffen Grampp, Mario Schiffer, Michael Wiesener, Johannes Schödel

Point-by-point response

Please find our replies to the comments from the reviewers highlighted in grey.

Reviewer #1:

Naas and colleagues provide an interesting report on the role of hypoxic expression of the kidney pathogenic gene MUC1. The authors used patients' derived primary tubular cells to study the role of hypoxia and hypoxia factor-1 stabilizers to study MUC1 expression. They report that both hypoxia and prolyl hydroxylases inhibitors increased MUC1 expression in a HIF-dependent way. Moreover, the gene risk variant associated with the development of chronic kidney disease was increasingly expressed under prolyl hydroxylase inhibitor treatment.

As pointed out above the study is interesting and well written. However, a few questions I would like the authors to address.

1. The authors compared incubation at 20.9 (control - normoxia?) and hypoxia on 1% O2. Certainly, 20.9 % O2 is not a physiological oxygen concentration. This would be rather closer to the 1 % than to the 20.9. Do the authors have any data at intermediate oxygen concentrations, e.g. 5 - 6 % O2? This may be relevant, particularly in view of Fig. 1 E, where they find MUC1 expression in their single cell analyses in the human kidney.

We thank the reviewer for evaluating our manuscript and providing very helpful comments.

The reviewer is right in that we used 20.9% O_2 (ambient oxygen level, "normoxia") as control conditions. We had declared this in the method section of the original manuscript. We agree with the reviewer that using ambient oxygen concentration as a comparator does not necessarily reflect the prevailing oxygen levels in human organs even under physiological conditions. However, measuring or predicting oxygen levels at a single cell resolution and at a certain time point in the kidney is still impossible. In order to support our findings on HIF-induced MUC1 expression from *in vitro* tissue culture experiments, we had resorted to the available single cell RNA-sequencing data set (doi.org/10.1186/s13073-022-01108-9), which provides valuable transcriptomic data from cells of acutely injured kidneys and control kidneys. Tubule cells of the AKI kidneys had a strong transcriptomic signature for hypoxia suggesting that in AKI oxygen levels are lower than in non-diseased kidneys which leads to activation of HIF. In line with our hypothesis that MUC1 is a HIF-target, expression levels of MUC1 were also elevated in the tubule cells from the injured kidneys.

As suggested by the reviewer, we now conducted new experiments exposing three independent cultures of primary tubular cells to different oxygen concentrations in a hypoxia chamber and measured MUC1 expression. Interestingly, we did not detect changes of MUC1 mRNA levels at 5% O_2 when compared to 20.9% O_2, although we did measure some stimulating effects on EGLN3 mRNA – a very sensitive HIF targets. This finding would suggest that more severe hypoxia is needed to activate MUC1 expression through HIF. Accordingly, levels of MUC1 do increase at 1% O_2 as measured in this new experiment and described in the manuscript earlier. Assuming that in some parts of the kidney the prevailing oxygen levels are comparable to 5% O_2, this finding would support the hypothesis that the cells experiencing more severe hypoxia (e.g. in ischemic insults) would be able to react by

upregulating MUC1. This would also be in line with the results from the single cell RNAseq data from human AKI as presented in the manuscript. We added the new data in new Supplemental Figure S6 and comment on this in the results section.

2. In most of their experiments, the effect of hypoxia was weaker than treatment with DMOG (PAD inhibitor). Again, the authors have experience with oxygen concentrations below 1 %? Moreover, Fig. 1 B show samples derived from two individuals. For both western blots comparing MUC1 and HIF-1 expression, it appears as if MUC1 expression is higher with moderately lower HIF-1α levels. Please, comment.

As outlined in the response to the first comment above, we conducted new experiments with exposing primary tubular cells isolated from kidneys of three independent individuals to various oxygen concentrations also including severe hypoxia at 0.1% O_2 (new Figure S6). At more severe hypoxia we measured a further increase of MUC1 mRNA and protein when compared to 5% or 1% O_2. This indicates that the increase of MUC1 expression depends on the prevailing O_2 concentration and would support a prominent role for this regulation in ischemic injuries in the kidney as outlined above.

This observation would also explain differences in MUC1 mRNA levels between hypoxia O_2 1% and pharmacological inhibition of PHDs by DMOG as highlighted by the reviewer. DMOG and very severe hypoxia (O_2 0.1%) also inhibit Factor-inhibiting HIF (FIH) which modulates HIF transcriptional activity. Thus, inhibition of FIH by DMOG might generate a greater HIF transcriptional response despite lower HIF protein levels. We used an established time interval of 16h for treating the cells with various stimuli including hypoxia, DMOG and clinically relevant PHD inhibitors to induce HIF protein and target gene expression. Cell-type specific kinetics of HIF-stabilization which will be counteracted by HIF-mediated induction of PHDs and reduction of drug efficiency over time can not be examined in this setting. Thus, the 16h time point integrates HIF-activity on MUC1 mRNA and protein expression as well as MUC1 degradation over the whole treatment period, but covers the presence of HIF-protein (not transcriptional activity) only at this specific time point. Thus, the results presented for MUC1 RNA and protein are in line with our hypothesis of MUC1 expression being under control of HIF in tubular cells.

We also would like to point out that we use precious primary tubular cell cultures derived from different donors which can differ in their cell composition (cultures include MUC1 expressing distal and non-MUC1 expressing proximal tubular cells) and the genetic background that could potentially influence the HIF-response.

3. Fig. 1 D with siRNA against HIF-1β indicates that there may be effects of DMOG other than through HIF signalling. With respect to HIF-1α siRNA, HIF-2α could be involved. In the discussion, the authors argue that PHD inhibitors are not selective. However, Daprodustat, one of the drugs to be used for HIF-2-dependent EPO expression, shows the strongest effect with respect to MUC expression. Do the authors have data with siRNA for HIF-1α and Daprodustat treatment?

a) HIF-siRNA: We thank the reviewer for this comment. We used siRNA against HIF-1β to corroborate the data on direct HIF-regulation of MUC1 derived from ChIP-seq (HIF-1α and HIF-1β) and HIF-1α knock-down experiments. In the primary cells, we generally achieve a knock-down efficiency of approximately 70-80% when using qPCR of HIF-1α or HIF-1β as a read-out. For primary tubular cells, which are very hard to transfect, this is a very good efficiency and it is in the range achieved by other groups for HIF-1α (doi.org/10.1074/jbc.M511408200). However, in this type of experiment we do not obtain a complete reduction or “knock-out” of HIF-1β protein and would expect some residual

capacity of HIF-α and HIF-1β to dimerize which would result in some residual induction of target genes (e.g. MUC1). This is what we observe in our experiments. We would like to point out that we repeated this experiment in cultures from tubule cell isolates from nine individuals with similar results.

However, we agree that from this experiment we cannot exclude HIF-independent effects of DMOG on MUC1 expression. Since we observe MUC1 induction also under other HIF-stabilizing conditions such as hypoxia or more PHD-specific inhibitors, we conclude that most of the effects observed on MUC1 induction is mediated directly via HIF. We also agree that HIF-2α might be involved in the regulation of MUC1, especially in cells from other tissues or tumors. However, protein expression of HIF-2α has not been detected in tubular cells in human or rodent kidney specimens in immunohistochemistry experiments. Again, in primary human tubular cells most of the increase of MUC1 mRNA levels upon HIF stabilization is abolished by HIF-1α knock-down (Figure 1D) indicating that this is the dominating isoform regulating MUC1 expression in this cell-type.

b) Daprodustat: all PHD-inhibitors used for clinical trials were designed to induce EPO production via stabilization of HIF-2α, but they are not selective for the HIF-α isoform. We used equimolar amounts of the compounds in our experiments to corroborate the effects of HIF-stabilization on MUC1 expression. These concentrations do not necessarily reflect concentrations of HIF-stabilizers observed in the blood or tissues of humans treated with these substances. Of note, in the study by Yeh et al. Daprodustat was more effective in stabilizing HIF (HIF-1α and HIF-2α) compared to other compounds tested in clinical trials. This probably reflects the differences in dosing observed in the clinical trials in humans (e.g. Daprodustat 1-24 mg/d vs Vadadustat 150 – 600mg/d). Thus, higher levels of MUC1 mRNA may simply reflect the more potent stabilizing effect of Daprodustat on HIF-levels compared to the other substances at this concentration.

In our cell model, DMOG and Daprodustat stabilize both HIF-isoforms as published earlier (doi.org/10.1016/j.kint.2023.03.035). To prove HIF-1α dependency of MUC1 induction under DMOG conditions we have performed HIF-1α kd in primary cell cultures from nine individuals, which resulted in an almost complete reduction of MUC1 mRNA induction. Although we did not perform HIF-1α knock-down experiments in cells treated with Daprodustat, from these experiments and the prominent role of HIF-1α in the tubular system as outlined above we expect that HIF-1α is the dominating isoform regulating MUC1 expression in PTC. Moreover, we performed dose-response experiments in PTC with different substances and observed that lowering the concentration of Daprodustat to about 10 µM results in comparable levels of MUC1 expression to 100 µM Vadadustat (**PBP_Figure 1**). This is in line with Daprodustat being a very potent HIF-stabilizer compared to the other compounds.

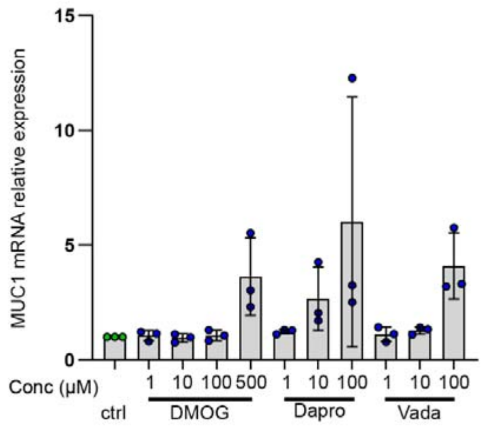

PBP_Figure 1: *Relative MUC1 mRNA expression levels in PTC. Normalized to expression values of HPRT mRNA and values from untreated cells. DMOG, Daprodustat (Dapro) and Vadadustat (Vada) were used at the indicated concentrations. Data is mean ±SD from three independent experiments.*

Minor point:
Fig. 2 is cut off at the end.

We apologize for this and corrected the figure.

Reviewer #2 (Comments to the Authors (Required)):

This manuscript highlighted the potential contribution of environmental factor on the course of kidney disease by modulating the expression of MUC1. The authors showed that stabilizing HIF-1a using different approaches induced MUC1 expression including the disease associated variants. Intriguingly, pharmacological compounds to stimulate EPO production by stabilizing HIF (MUC1 upstream regulator) in the diseased kidney of CKD patients before and during dialysis have been clinically approved. Thus, this study forewarns that using HIF stabilizing agents should be used with great caution in ADTKD MUC1 patients as these drugs may aggravate kidney disease by enhancing MUC1 expression including the deleterious variants.

1.MUC1 has been shown to be induced and play a key role in both human and mouse kidney proximal tubule cells following ischemia reperfusion injury (PMID: 26739894 and 34151589). The authors should consider in their introduction and discussion sections the role of MUC1 in proximal tubule where damage is most significant following the ischemia (hypoxia) injury protocol.

Thank you for reviewing our manuscript and the comment on MUC1 expression in the proximal tubule. The single cell RNAseq data from Hinze et al. (doi.org/10.1186/s13073-022-01108-9; presented in Supplemental Figure S3) confirm the above-mentioned observation of MUC1 induction upon acute injury. Therefore, we have included comments on the expression and role of MUC1 in the proximal tubule in the introduction and expanded the discussion section on this topic.

2.Used anti-HIF-1a antibody (Cay1000062421) in the study needs to be validated as the authors considered the 130 kDa band represents HIF-1a, while the antibody's company website considered the 130 kDa band as nonspecific band and the corrected band size for HIF-1a is ~100 kDa. One blot was cut just below 130 kDa and it's hard to see if there is any band ~100 kDa.

We very much agree with the reviewer that antibodies used in immunoblotting experiments need to be validated. First, we want to apologize, but the correct ID number of the antibody used is Cay10006421 which we now corrected in the revised manuscript. We had validated the anti-HIF-1α antibody (Cay10006421) extensively in previous work (doi.org/10.1074/jbc.RA119.009827, Supplemental Figure 3 and doi.org/10.1016/j.jbc.2022.101699 Supplemental Figure 3) by using siRNA against HIF-1α in four different cell lines (for an example please see **PBP_Figure 2**). This revealed a specific band at about 130 kDa, which corresponds to the signal that is gained by hypoxic exposure and lost upon HIF-1α knock-down. In addition, we have also conducted siRNA mediated knock-down of HIF-1α in primary tubular cells, which we used for this study, and confirmed specificity in this cell type (**PBP_Figure 3**). Of note, in this cell-type we do not detect any unspecific band between 130 and 150 kDa as described for the Cos cells published on the company's website. Thus, we are very confident that this antibody detects HIF-1α protein at the right size in immunoblots. To provide more transparency of our results from immunoblot experiments, we now include uncropped blots of all immunoblot experiments in the supplemental files. We have added a comment on our antibody validation and the references in the methods section.

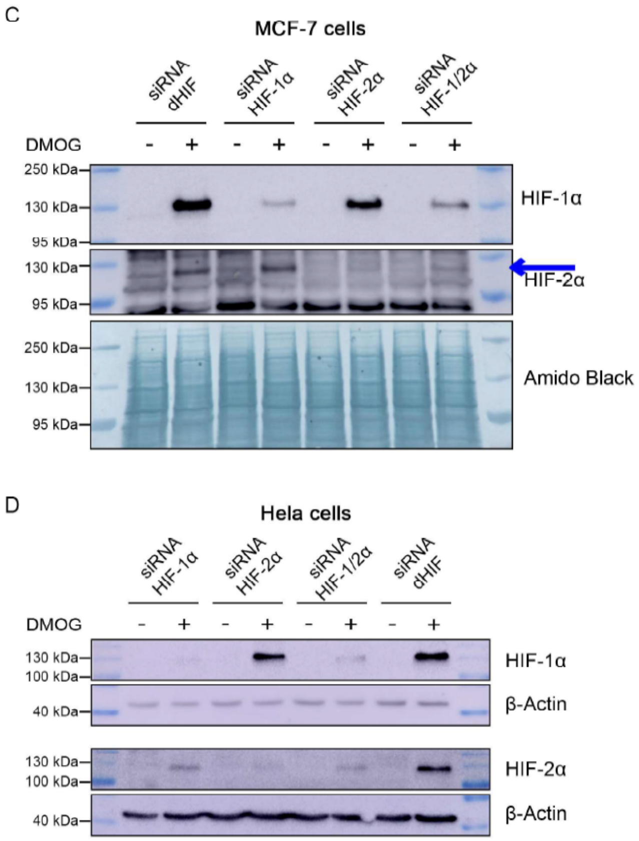

PBP_Figure 2: *Validation of HIF antibodies (for HIF-1a Cay10006421 was used). dHIF: control siRNA. DMOG was used at 1 mM to stabilize HIF. From Supplemental Figure 3, Protze J. et al. doi.org/10.1016/j.jbc.2022.101699.*

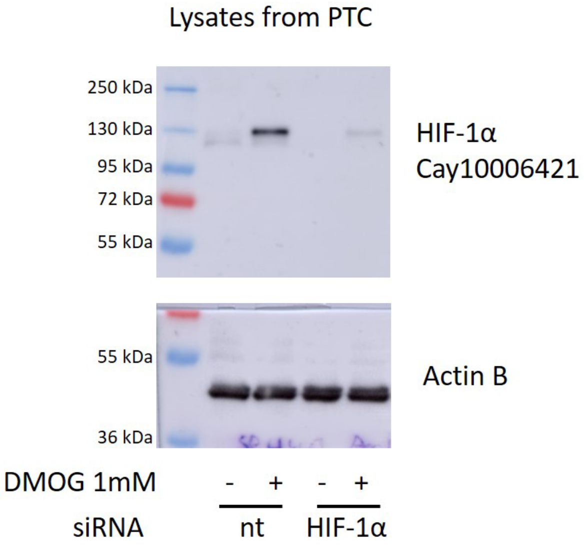

PBP_Figure 3: *Validation of anti- HIF-1a antibody Cay10006421 in primary renal tubular cells (PTC) used in this study. Nt: non-targeting control siRNA. DMOG was used at 1 mM for 16h to stabilize HIF.*

3.Statistical analysis needs to be revised again as t-test can be used to compare two different groups only, while for three or more groups (the case of most figures) ANOVA need to be performed followed by pairwise comparison.

We thank the reviewer for this comment. We have performed ANOVA testing for the relevant data and revised the figures 1D, 2D, S1 and S4C accordingly.

May 31, 2023

RE: Life Science Alliance Manuscript #LSA-2023-02078-TR

Dr. Johannes Schödel
Universitätsklinikum Erlangen
Department of Nephrology and Hypertension
Friedrich-Alexander-University Erlangen-Nuremberg
Ulmenweg 18
Erlangen D-91054
Germany

Dear Dr. Schödel,

Thank you for submitting your revised manuscript entitled "Hypoxia controls expression of kidney-pathogenic MUC1 variants". We would be happy to publish your paper in Life Science Alliance pending final revisions necessary to meet our formatting guidelines.

Along with points mentioned below, please tend to the following:

-please upload your supplementary figures as separate single files
-please add the ORCID ID for the corresponding author-you should have received instructions on how to do so

If you are planning a press release on your work, please inform us immediately to allow informing our production team and scheduling a release date.

LSA now encourages authors to provide a 30-60 second video where the study is briefly explained. We will use these videos on social media to promote the published paper and the presenting author (for examples, see https://twitter.com/LSAjournal/timelines/1437405065917124608). Corresponding or first-authors are welcome to submit the video. Please submit only one video per manuscript. The video can be emailed to contact@life-science-alliance.org

To upload the final version of your manuscript, please log in to your account: https://lsa.msubmit.net/cgi-bin/main.plex
You will be guided to complete the submission of your revised manuscript and to fill in all necessary information. Please get in touch in case you do not know or remember your login name.

To avoid unnecessary delays in the acceptance and publication of your paper, please read the following information carefully.

A. FINAL FILES:

These items are required for acceptance.

-- An editable version of the final text (.DOC or .DOCX) is needed for copyediting (no PDFs).

-- High-resolution figure, supplementary figure and video files uploaded as individual files: See our detailed guidelines for preparing your production-ready images, https://www.life-science-alliance.org/authors

-- Summary blurb (enter in submission system): A short text summarizing in a single sentence the study (max. 200 characters including spaces). This text is used in conjunction with the titles of papers, hence should be informative and complementary to the title. It should describe the context and significance of the findings for a general readership; it should be written in the present tense and refer to the work in the third person. Author names should not be mentioned.

B. MANUSCRIPT ORGANIZATION AND FORMATTING:

Full guidelines are available on our Instructions for Authors page, https://www.life-science-alliance.org/authors

We encourage our authors to provide original source data, particularly uncropped/-processed electrophoretic blots and spreadsheets for the main figures of the manuscript. If you would like to add source data, we would welcome one PDF/Excel-file per figure for this information. These files will be linked online as supplementary "Source Data" files.

**Submission of a paper that does not conform to Life Science Alliance guidelines will delay the acceptance of your

manuscript.**

It is Life Science Alliance policy that if requested, original data images must be made available to the editors. Failure to provide original images upon request will result in unavoidable delays in publication. Please ensure that you have access to all original data images prior to final submission.

The license to publish form must be signed before your manuscript can be sent to production. A link to the electronic license to publish form will be sent to the corresponding author only. Please take a moment to check your funder requirements.

Reviews, decision letters, and point-by-point responses associated with peer-review at Life Science Alliance will be published online, alongside the manuscript. If you do want to opt out of having the reviewer reports and your point-by-point responses displayed, please let us know immediately.

Thank you for your attention to these final processing requirements. Please revise and format the manuscript and upload materials within 7 days.

Thank you for this interesting contribution, we look forward to publishing your paper in Life Science Alliance.

Sincerely,

Novella Guidi, PhD
Scientific Editor
Life Science Alliance

Reviewer #1 (Comments to the Authors (Required)):

Thank you for your response - I have no further questions

Reviewer #2 (Comments to the Authors (Required)):

All concerns have been addressed in a professional and clear way.

June 1, 2023

RE: Life Science Alliance Manuscript #LSA-2023-02078-TRR

Dr. Johannes Schödel
Universitätsklinikum Erlangen
Department of Nephrology and Hypertension
Friedrich-Alexander-University Erlangen-Nuremberg
Ulmenweg 18
Erlangen D-91054
Germany

Dear Dr. Schödel,

Thank you for submitting your Research Article entitled "Hypoxia controls expression of kidney-pathogenic MUC1 variants". It is a pleasure to let you know that your manuscript is now accepted for publication in Life Science Alliance. Congratulations on this interesting work.

The final published version of your manuscript will be deposited by us to PubMed Central upon online publication.

Your manuscript will now progress through copyediting and proofing. It is journal policy that authors provide original data upon request.

Reviews, decision letters, and point-by-point responses associated with peer-review at Life Science Alliance will be published online, alongside the manuscript. If you do want to opt out of having the reviewer reports and your point-by-point responses displayed, please let us know immediately.

IMPORTANT: If you will be unreachable at any time, please provide us with the email address of an alternate author. Failure to respond to routine queries may lead to unavoidable delays in publication.

Scheduling details will be available from our production department. You will receive proofs shortly before the publication date. Only essential corrections can be made at the proof stage so if there are any minor final changes you wish to make to the manuscript, please let the journal office know now.

DISTRIBUTION OF MATERIALS:
Authors are required to distribute freely any materials used in experiments published in Life Science Alliance. Authors are encouraged to deposit materials used in their studies to the appropriate repositories for distribution to researchers.

You can contact the journal office with any questions, contact@life-science-alliance.org

Again, congratulations on a very nice paper. I hope you found the review process to be constructive and are pleased with how the manuscript was handled editorially. We look forward to future exciting submissions from your lab.

Sincerely,

Novella Guidi, PhD
Scientific Editor
Life Science Alliance
